# In situ observation of deformation processes in nanocrystalline face-centered cubic metals

**DOI:** 10.3762/bjnano.7.50

**Published:** 2016-04-19

**Authors:** Aaron Kobler, Christian Brandl, Horst Hahn, Christian Kübel

**Affiliations:** 1 Institute of Nanotechnology (INT), Karlsruhe Institute of Technology (KIT), Hermann-von-Helmholtz-Platz 1, 76344 Eggenstein-Leopoldshafen, Germany; 2 Joint Research Laboratory Nanomaterials (KIT and TUD), Technische Universität Darmstadt (TUD), Jovanka-Bontschits-Straße 2, 64287 Darmstadt, Germany; 3 Institute for Applied Materials (IAM-WBM), Karlsruhe Institute of Technology (KIT), Hermann-von-Helmholtz-Platz 1, 76344 Eggenstein-Leopoldshafen, Germany,; 4 Karlsruhe Nano Micro Facility (KNMF), Karlsruhe Institute of Technology (KIT), Hermann-von-Helmholtz-Platz 1, 76344 Eggenstein-Leopoldshafen, Germany

**Keywords:** ACOM-STEM, deformation mechanisms, in situ straining, nanocrystalline metals, orientation mapping, quantitative crystallographic analysis

## Abstract

The atomistic mechanisms active during plastic deformation of nanocrystalline metals are still a subject of controversy. The recently developed approach of combining automated crystal orientation mapping (ACOM) and in situ straining inside a transmission electron microscope was applied to study the deformation of nanocrystalline Pd*_x_*Au_1−_*_x_* thin films. This combination enables direct imaging of simultaneously occurring plastic deformation processes in one experiment, such as grain boundary motion, twin activity and grain rotation. Large-angle grain rotations with ≈39° and ≈60° occur and can be related to twin formation, twin migration and twin–twin interaction as a result of partial dislocation activity. Furthermore, plastic deformation in nanocrystalline thin films was found to be partially reversible upon rupture of the film. In conclusion, conventional deformation mechanisms are still active in nanocrystalline metals but with different weighting as compared with conventional materials with coarser grains.

## Introduction

**Figure 1:**
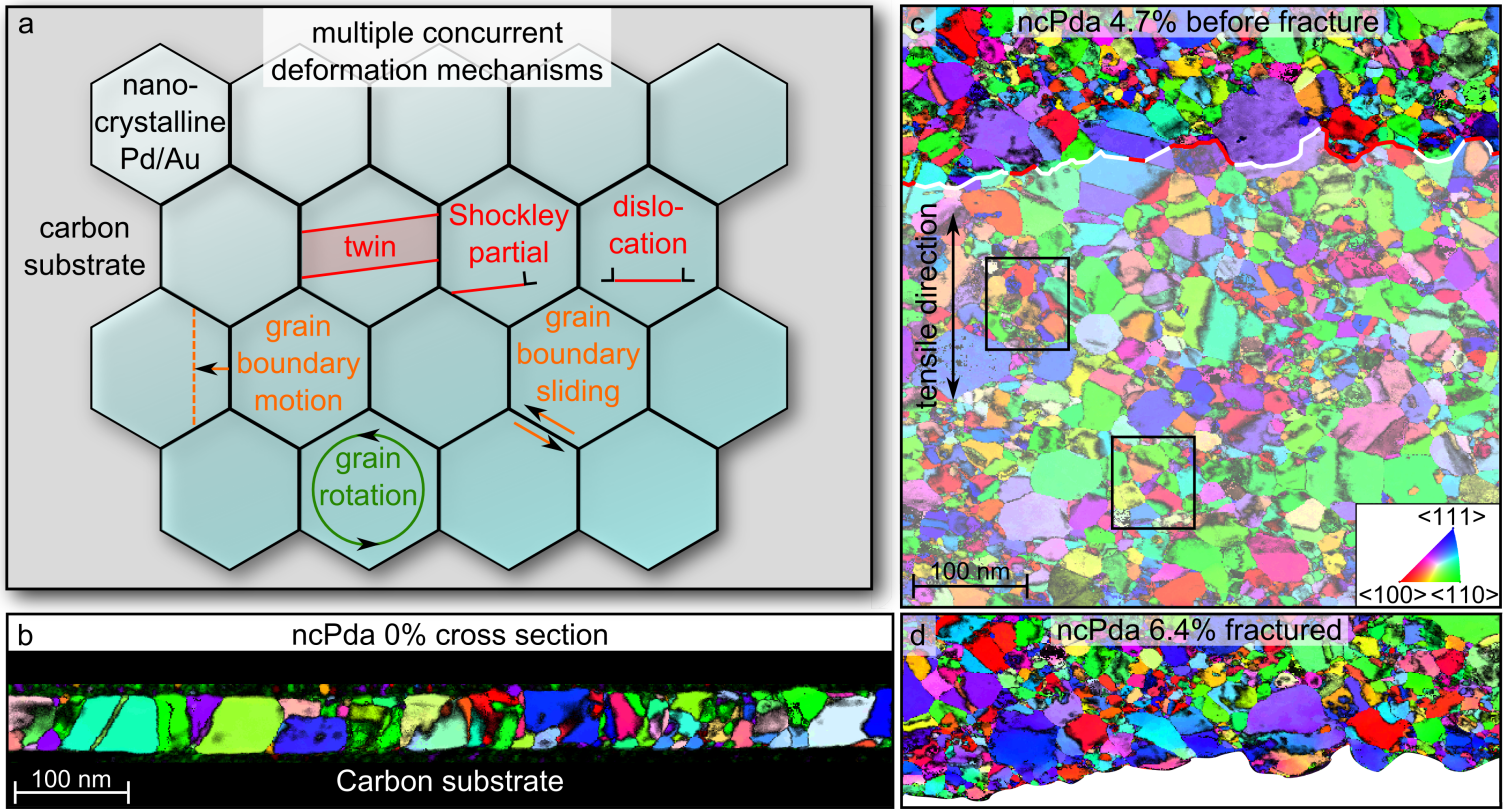
(a) Sketch showing multiple concurrent deformation mechanisms. (b) ACOM-STEM orientation maps overlaid with the reliability and the cross-correlation index of a cross-section of ncPda at 0% strain. (c,d) ACOM-STEM orientation maps overlaid with the reliability of the fracture morphology of ncPda before (c) and after fracture (d). The crack line of (d) is overlaid in (c) to reveal the crack behavior. Red parts of the crack line indicate fracture along grains boundaries and white lines indicate cracks passing through grains. The projection direction is along the tensile direction and the color code is given in (c). The black boxes in (c) indicate the areas of the detailed analysis in Figure 2 and Figure 3.

Nanocrystalline (NC) metals and alloys with grain size below 100 nm exhibit outstanding mechanical properties, in particular, superior hardness, strength and fatigue properties as compared to their coarse grained counterparts [1–5]. Modified or even unexpected deformation mechanisms are ascribed to the increasing influence of grain boundaries (GBs) on mechanical properties [3–4,6–20]. GB-mediated deformation mechanisms, such as GB sliding and migration, grain growth and rotation [21] have been discussed (Figure 1a). Dislocation-mediated plasticity in the grain interior is reported to be based on dislocation nucleation at GBs, propagation and eventual absorption in the surrounding GB network, as well as twin nucleation, migration and detwinning (Figure 1a). Such twin activity and dislocation-driven grain rotation can still be attributed to dislocation formation, propagation and adsorption [13,22]. The understanding of the active deformation mechanisms is essential to support the application of NC metals, for example, in microelectrical mechanical systems (MEMS) [23], hydrogen storage materials [24], radiation-resistant materials for nuclear reactors [25], applications for wear and corrosion protection [6,26] and for flexible electrical components [27].

Beside simulations [12–14,17,28–29], only few experimental methods are capable to measure in situ structural signatures of the deformation mechanisms in NC metals during mechanical deformation. X-ray diffraction (XRD) is one of the experimental methods [4,11,26,30]. However, XRD cannot directly image and spatially resolve deformation processes (e.g., grain growth or twinning); it measures the global structural signatures of the diffracting volume, which is encoded in the diffraction pattern and peak profile. For a local analysis, NC metals are traditionally investigated using bright/dark field transmission electron microscopy (BF/DF-TEM) [31–33] or high resolution TEM (HRTEM) [34]. In situ BF/DF-TEM deformation experiments are even more challenging to uniquely interpret local changes of nanometer-sized grains because of varying image contrast due to bending or tilting of the whole sample as well as lattice (grain) rotation within the sample during testing. The electron-transparent thin films necessary for TEM might also show more surface effects in comparison to bulk techniques. On the micrometer scale and for ultra-fine grained materials, local crystal orientations at the sample surface can be quantitatively tracked in a scanning electron microscope (SEM) by electron back scatter diffraction (EBSD) [35]. However, NC metals are beyond the spatial resolution limits of EBSD (>20 nm) [36].

Here, we demonstrate a combination of automated crystal orientation mapping (ACOM) in scanning TEM (STEM) modus with in situ straining [37–39] of NC metals to follow the GB-mediated processes (GB sliding and grain growth) and dislocation-mediated intragranular formation of twins to provide real space evidence for the active deformation mechanisms. Contrary to conventional TEM investigations, the crystal orientation of all grains within the investigated area are tracked quantitatively, which allows sample bending/tilting effects to be separated from real local crystallographic changes [38].

In situ ACOM-STEM is applied to magnetron-sputtered NC thin film samples exhibiting a predominately columnar grain structure. In the following, we concentrate on results from an annealed NC Pd (ncPda) thin film with ≈50 nm thickness supported by a thin (≈20 nm) carbon film (Figure 1b). An additional experiment using an annealed NC AuPd (ncAuPda) thin film was also conducted and the results are compared with the ncPda results at the end of the paper.

In this study, a distinction between crystallites and grains is made. Crystallites are defined as the smallest volume with one crystal orientation within a disorientation tolerance of 3° and grains are either single crystallites or multiple crystallites connected by twin boundaries.

## Results and Discussion

All presented ACOM-STEM orientation maps (color coding according to the inverse pole figure, inset in Figure 1c) are superimposed with the reliability values in black, which constitute a measure for the unambiguousness of the orientation determination during the orientation assignment to the diffraction pattern. The evolution of the local orientation is tracked in a tensile experiment until fracture (Figure 1d). This procedure allows the assignment of the fracture site to the microstructural features (i.e., grain boundaries) (Figure 1c). The direct overlay reveals that the crack behavior is a combination of intragranular fracture with the crack path through a crystallite (white lines in Figure 1c) and intergranular fracture with the crack path along grain boundaries (red lines in Figure 1c). The observation of grain growth, fragmentation and recrystallization next to the crack path (Figure 1c,d) suggests that the deformation is both a result of intragranular deformation (related to dislocation-mediated processes in grains) and intergranular deformation by GB-mediated deformation mechanisms.

**Figure 2:**
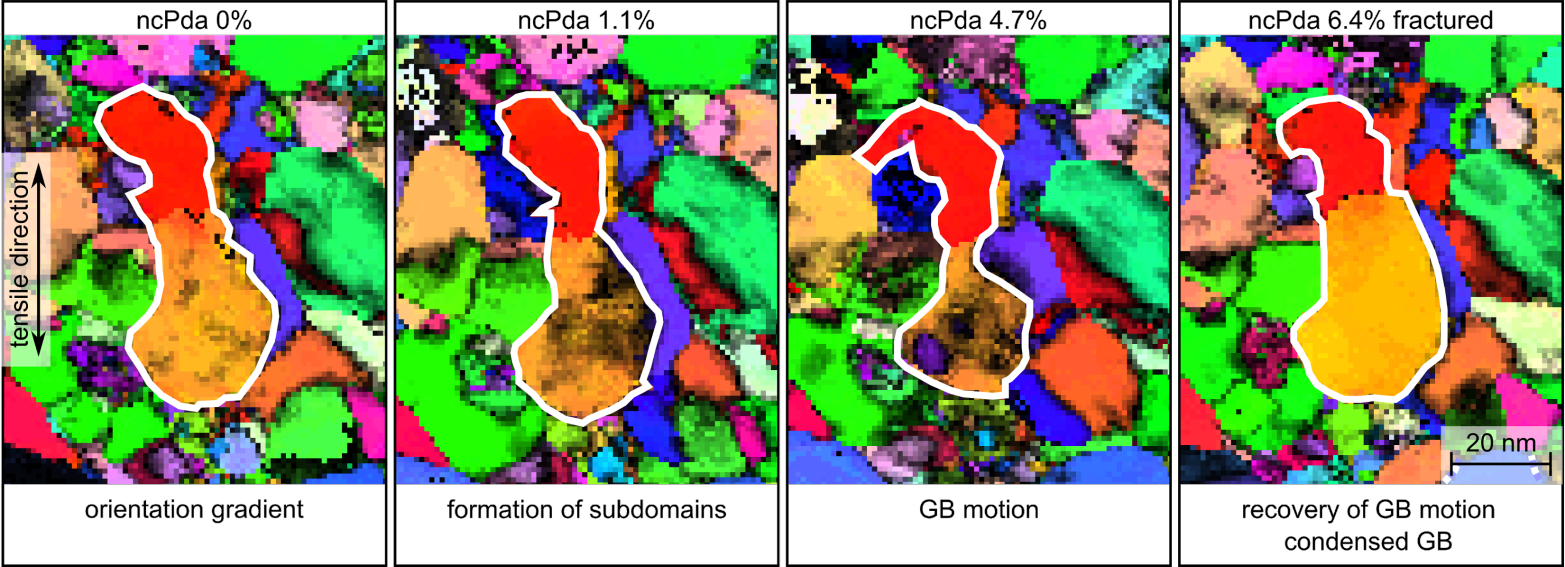
Grain fragmentation of the center grain (white boundary) during the tensile deformation (0–4.7% strain) of ncPda and grain recovery after fracture (6.4% strain) of the sample. The ACOM-STEM orientation maps overlaid with the reliability are shown for selected straining states with the projection direction along the tensile direction and the color code is given in Figure 1c.

Focusing on a single grain level, Figure 2 shows details of the orientation map in Figure 1c for selected straining states to visualize the grain’s structural changes during tensile loading and subsequent unloading by fracture. The overall size of the encircled grain (white line) continuously decreases during the tensile loading until 4.7%, directly showing GB motion during mechanical loading. However, upon fracture, the initial grain shape and grain size is almost recovered. The neighboring grains on the lower right hand side indicated in purple/blue and orange show similar recovery processes. The recovery of the grain shape indicates that the GB motion is the result of an external driving force rather than the reduction of GB area as in the case of conventional grain growth. Moreover, the pre-existing orientation gradient (orange to red color in Figure 2; lattice perturbations along a ≈15 nm section across the boundary shown in Supporting Information File 1, Figure S11a) at 0% strain is subsequently condensed (red and orange crystallite at 1.1% strain in Figure 2, ≈15° disorientation Supporting Information File 1, Figure S11b). The high Schmid factor (≈0.5) of the orange crystallite (Supporting Information File 1, Figure S8) in conjunction with the decreasing reliability (amount of black pixels increases) from 0% to 4.7% strain is consistent with the creation and accumulation of mobile and/or sessile dislocations in the grain during the tensile load [40–42], which can condense around the boundary and can leave the orange crystallite with reduced defect concentration in the grain upon fracture (see the disorientation to mean orientation plot, Supporting Information File 1, Figure S11a). Previous in situ X-ray diffraction studies revealed reversible diffraction peak broadening in a loading–unloading cycle in NC Ni, which indicates the suppression of a build-up of a residual dislocation network during deformation in the nanograins [30]. Here the accumulation of defects into a sharp small angle GB (Figure 2, 6.4%) upon unloading by fracture is, however, consistent with the accumulation of a residual dislocation network in the nanograin during deformation, which condenses into a small angle grain boundary. Moreover, the reversible GB motion observed here can also explain the previously reported extraordinary strain recovery in NC Al and NC Au thin films [43].

**Figure 3:**
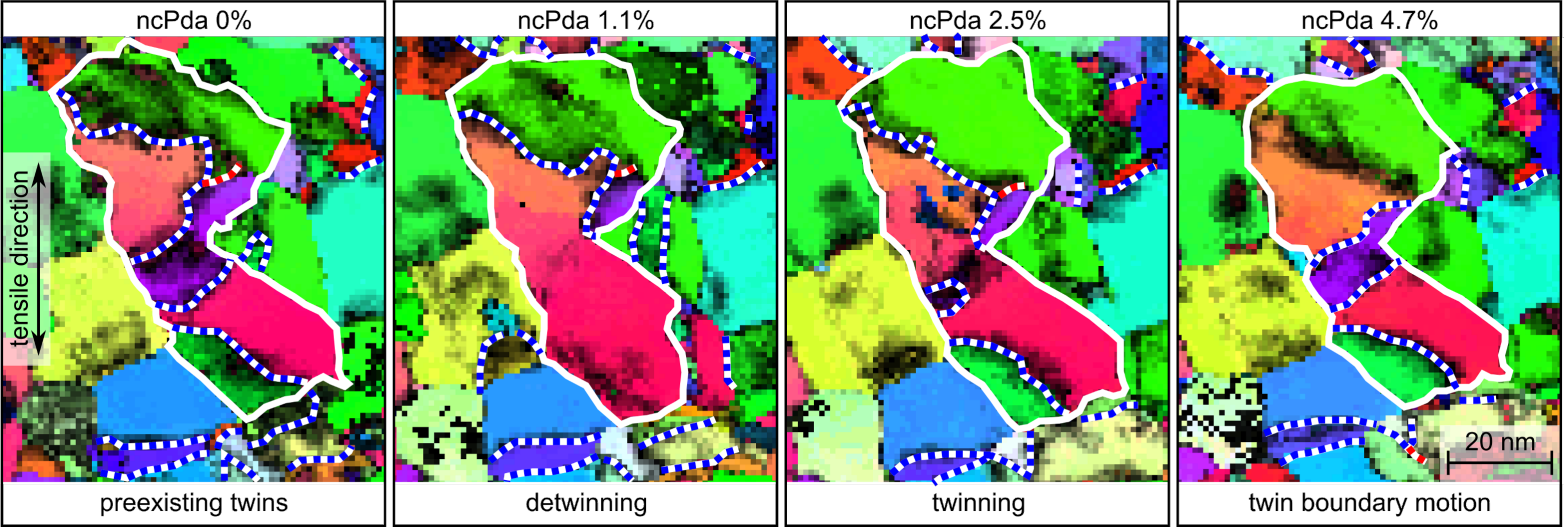
Twinning, twin boundary migration and detwinning of the center grain (white boundary) during the tensile deformation (0-4.7% strain) of ncPda. The ACOM-STEM orientation maps overlaid with the reliability are shown for selected straining states with the projection direction along the tensile direction and the color code is given in Figure 1c. The Σ3 disorientations are marked with white/blue dashed lines and the Σ9 disorientations with white/red dashed lines.

Figure 3 shows a sequence of detwinning (0–1.1% purple red grain), twin nucleation (1.1–2.5%) and twin growth (2.5–4.7%). The twin activity is accompanied by substantial structural changes in the surrounding grains (e.g., white/grey crystallite at the bottom grows with straining). In general, the twin activity correlates with local plastic strain manifested in the structural changes. Since Shockley partial dislocations of the fcc lattice have the same Burgers vectors as the intrinsic crystallographic structure elements of twin (Σ3) boundaries [44], the twin motion indicates the operation of Shockley partial dislocations entering the grain from the boundary or moving along the preexisting twin planes.

**Figure 4:**
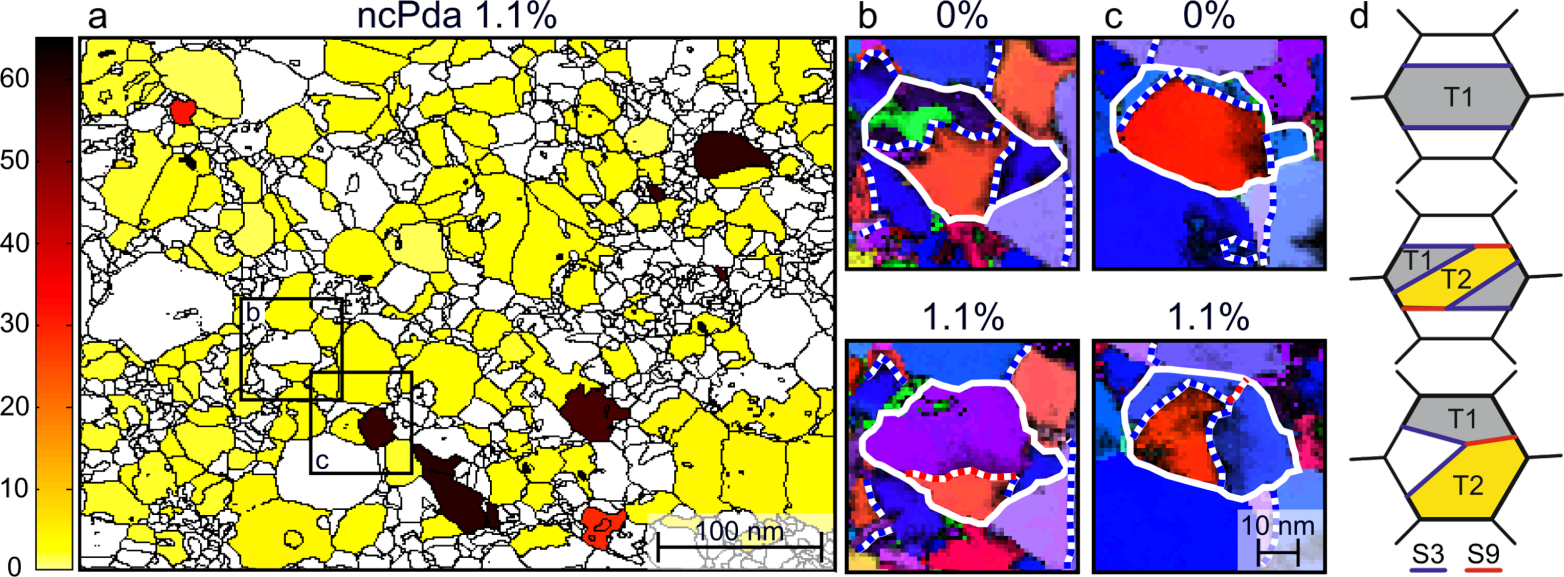
Analysis of the crystallite rotations in the range 0–65° for ncPda: (a) Crystallite boundary map overlaid with the rotation of selected and tracked crystallites for the 1.1% strain state. The color code is displayed on the left. (b,c) ACOM-STEM orientation maps of selected areas marked in (a). Σ3 disorientations marked with white/blue dashed lines and Σ9 disorientations marked with white/red dashed lines; the white boundary outlines the grain of interest. Projection direction: normal to the paper plane; color code is given in Figure 1c. (b) Details of a twin boundary activity. (c) Details of a grain that shows the creation of a triple line with two Σ3 and one Σ9 disorientation. (d) Schematic of the formation of Σ3 and Σ9 disorientations. The twin domains are labeled as grey/yellow areas, and the twin boundaries are indicated by the blue solid lines. The combination of two distinct twin variants (T1 in grey and T2 in yellow) results in a triple lines of two Σ3 (blue) and one Σ9 (red) boundary, which is equivalent to a chain of twin disorientation [46].

**Figure 5:**
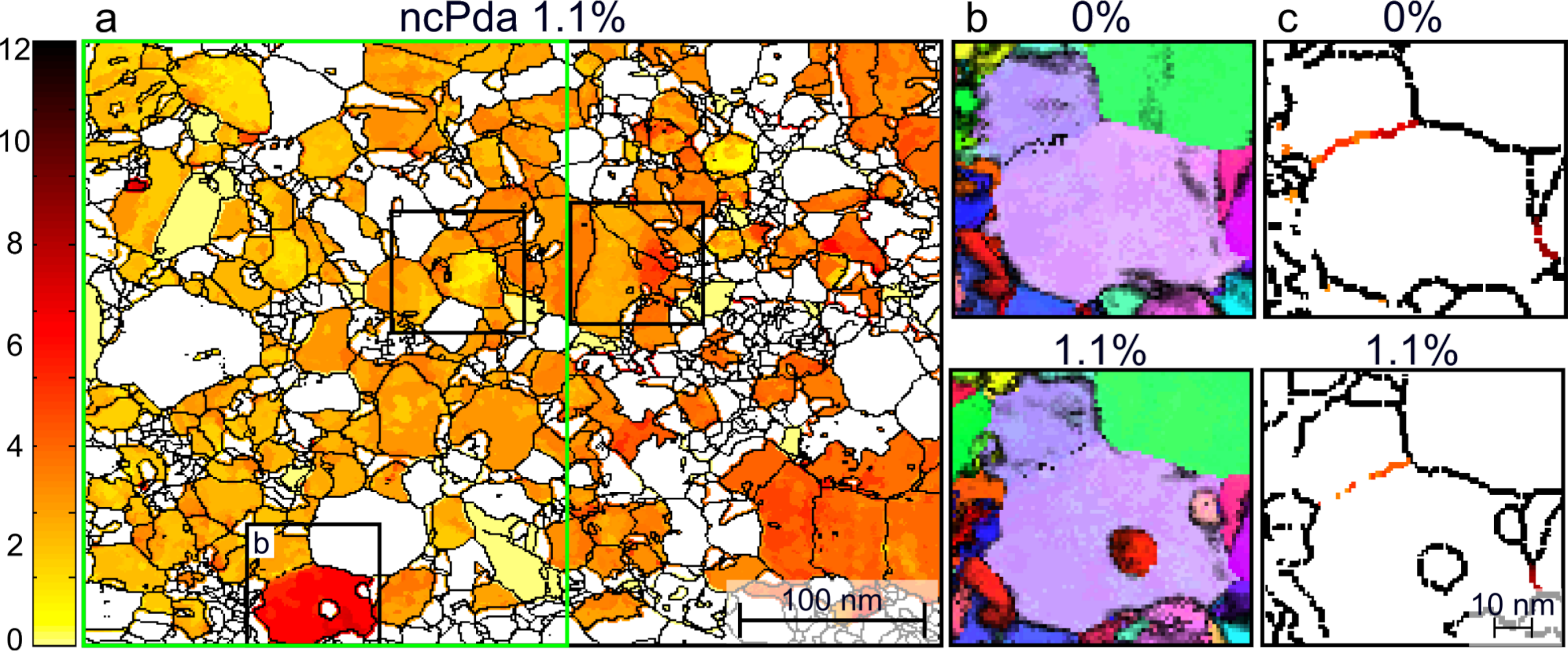
Analysis of the crystallite rotations in the range 0–12° for ncPda: (a) Crystallite boundary map of ncPda overlaid with the rotation of each pixel of individually tracked crystallites for the 1.1% strain state. The color code for the overlaid rotation of the crystallites is displayed on the left. (The green frame is the area which is evaluated in Figure 6 and additional black boxes indicate local deviations from the sample bending) (b) ACOM-STEM orientation maps of a selected area marked in (a). Projection direction: along the tensile direction; color code is given in Figure 1c. (c) Boundary disorientation plot of (b) with color coding according to (a).

The twin activity associated with heterogeneous distribution of the plastic strain indicates the necessity of accommodating processes to suppress fracture by local plastic strain incompatibilities. Grain-to-grain level ACOM-STEM measurements are used to follow the change of lattice orientation relative to the tensile loading direction. The absolute orientation change of the individually tracked grains through the straining series with respect to the initial state is displayed in a rotation map (Figure 4a and Figure 5a). This map allows the heterogeneous deformation in NC metals to be separated from an overall sample bending or tilting. Figure 4a shows the crystallite rotations in the range 0–65° and clearly indicates individual grains with ≈39° and ≈60° rotations (red and black), which are consistent with Σ3 and Σ9 misorientation relations, respectively. Additionally, Figure 5a highlights the crystallite rotations in the range 0–12° and uncovers the heterogeneous deformation in the small angle rotations, which are accommodated along GBs. Our observations are in line with previous work on NC Pt [34] and NC Ni [45].

Focusing on the large angle crystallite rotations (0–65°), Figure 4b shows the coalescence of two Σ3 boundaries (white/blue dashed lines at 0%) to one Σ9 boundary (white/red dashed line at 1.1%; a detailed analysis is shown in Supporting Information File 1, Figure S12). In Figure 4c, another example for twin boundary movement is shown. The triple line at 1.1% strain formed by two twin boundaries (white/blue dashed lines) and one Σ9 boundary (red/blue dashed line) originating from a grain boundary at 0% strain and the migration of the triple line half way through the grain during tensile loading. Since the Σ9 misorientation can be generated by a sequence of two distinct twin variants [46–47], Figure 4d illustrates schematically possible twin morphologies in one grain: the upper schematic shows a single twin domain (T1) in a grain. The twin-in-twin morphology, a secondary twin (T2) developed in the primary twin domain (T1), is shown in the middle schematic. The lower schematic shows the coalescence of two twin boundaries of two distinct twin variant disorientations, T1 and T2, creating a triple line with a Σ9 boundary. Generalized, the observed Σ9 boundary in the NC system is a signature of subsequent or multiple twinning in a grain (as shown in Figure 4d, middle and lower schematic), and the nucleation as well as migration of Σ9 boundaries is a result of two different sets of lattice partial dislocation motions associate with the coalesced Σ3 boundaries [22].

Concentrating now on the small angle crystallite rotations (0–12°), Figure 5a displays an overall bending of the thin TEM film during deformation that appears as a rotation gradient (left to right). Figure 5a shows additional local deviations from the sample bending, which appear as local, distinct rotations within the crystallites and in the vicinity of the grain boundaries (examples indicated by black boxes). A mismatch of the relative lattice rotation of neighboring grains needs to be accommodated as a shear deformation across the GB between the crystallites. The small angle rotation discontinuity at the grain boundary directly shows the operation of GB deformation processes, such as GB sliding and/or shear coupled GB motion.

The small angle crystallite rotation highlighted in Figure 5b,c even shows the partial coalescence of two crystallites with an initial disorientation of ≈4–7° at 0% strain. During deformation (at 1.1% strain), the disorientation between the crystallites partially disappears predominantly by the ≈4° rotation of the lower grain relative to its environment (Figure 5c). Grain boundary migration can be excluded, since a residual disorientation remains approximately at the same location of the former small angle grain boundary. The reduction of the disorientation is, therefore, associated with dislocations pushed from the small angle grain boundary into the crystallite or dislocations from the crystallite annihilate the small angle grain boundary dislocations. Both processes effectively reduce the dislocation content of the small angle grain boundary.

**Figure 6:**
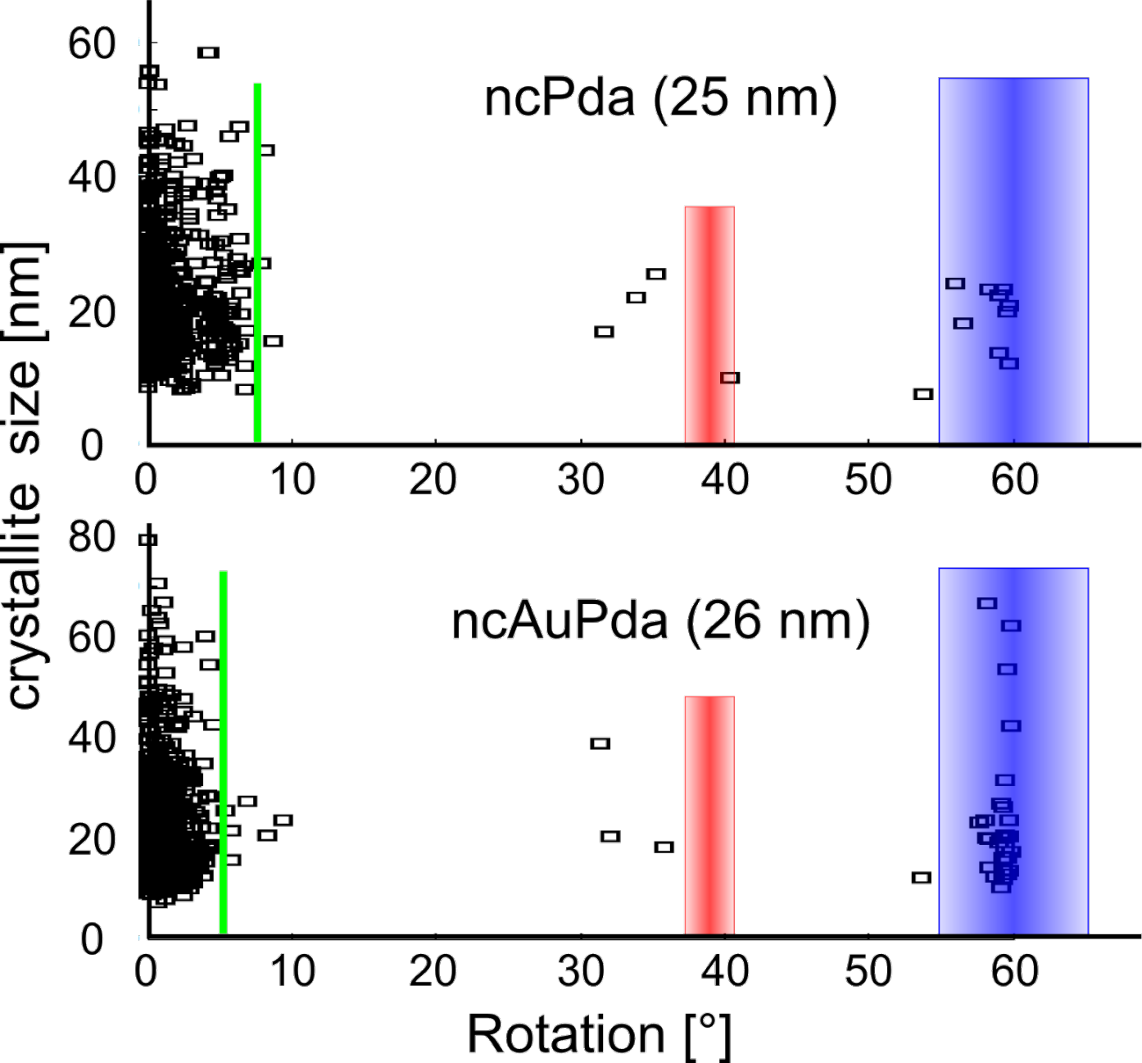
Crystalllite size versus lattice rotation plot of all tracked crystallites: (a) ncPda (*d*_A_ = 25 nm) (the green area of Figure 5a is evaluated), (b) ncAuPda (*d*_A_ = 26 nm). The green line indicates the rotation due to sample bending during straining. The Σ9 (39 ± 1.7°) and Σ3 (60 ± 5°) disorientations are marked in red and blue, respectively.

Figure 6 shows the signatures of the previously spatially resolved crystallite rotations versus the crystallite size for the tracked crystallites of ncPda (*d*_A_ = 25 nm) and ncAuPda (*d*_A_ = 26 nm). Both systems have a multimodal distribution with peaks in the small angle regime (<10°), a ≈39° regime (Σ9-rotation), and a ≈60° twin regime (Σ3-rotation) (a similar distribution has been found in two other Pd–Au systems not shown here). The small angle regime is a convolution of film bending (indicated by the green line in the histogram) and crystallite rotation due to heterogeneous intergranular deformation, which is accommodated by GB-mediated processes (Figure 5). Twin and multiple twin activity (Σ3 and Σ9 activity) are observed in all Pd–Au alloys investigated here. No significantly different behavior (within the statistical limitations) was observed for the different alloy systems, although molecular dynamics simulations suggest different dislocation and stacking fault densities with increasing strain as well as different grain boundary migration behavior for different alloy compositions [28,48]. In situ XRD studies also showed evidence on the concentration-dependent deformation behavior in the Pd–Au alloy [49]. Despite the good grain statistics of ACOM-TEM in comparison to HRTEM, synchrotron-based in situ XRD studies offer better temporal resolution in comparison to ACOM-TEM with far better grain statistics. Both good statistics and temporal resolution are necessary to reveal small differences in the deformation mechanism with the alloy content. With ACOM-TEM, the multiple concurrent mechanisms in all alloy systems are apparent. The twin activity does not reveal any statistically significant crystallite size dependence within the grain size distribution of the samples. This is a contradiction to previous observations on a grain size dependence of twinning in NC fcc metals [20]. Changing the alloy content and the sputter parameter can also increase the internal stresses and eventually the dislocation and twin density [50–51]. Besides the dislocation content and growth twins, the grain size and structure as well as texture at the beginning of straining are expected to influence the strength, the detailed interplay and transition of multiple concurrent mechanisms with increasing strain. The two presented systems were comparable in grain size and twin density (Supporting Information File 1, Figure S7, S18, Table S1), which allows a direct comparison. However, more measurements and better grain statistics are needed and may eventually reveal the transition and strength of the active deformation mechanisms in nanocrystalline material.

## Conclusion

In summary, the utilization of ACOM-STEM in combination with in situ straining of ncPda and ncAuPda inside the TEM revealed the spatial–temporal interaction of intergranular and intragranular proccesses, which are operated by GB-mediated and dislocation-mediated deformation mechanisms. Moreover, the insights gained here reveal that GB and dislocation-based deformation mechanisms are still operational at grain sizes as low as 10 nm (number averaged) in the Pd–Au system. Our observations question the absolute statements on the exclusive operation of one class of deformation mechanisms at the nanoscale and favor the explanation of multiple concurrent mechanisms and with different overall weighting between them compared to their coarse-grained counter parts.

## Experimental

Metal thin films were deposited by radio frequency (RF) magnetron sputtering using 2" diameter planar targets with 99.95% purity. TEM grids (holycarbon R2/1 + 2 nm C, Quantifoil) were used as a substrate. The nominal thickness between the holes is 22 nm. Pd was sputtered in 5 cycles of 50.38 s at 60 W constant sputtering power under a working gas pressure (Ar) of 0.005 mbar at room temperature. Au (72 atom %) Pd (28 atom %) was sputtered for 102.5 s at 60 W constant sputtering power (for both Pd and Au) under a working gas pressure (Ar) of 0.0055 mbar at room temperature. The root mean squared (RMS) roughness between the holes was measured by AFM to be 1.3 nm for the ncPda film (Supporting Information File 1, Figure S2). The thickness of the films (ncPda ≈50 nm; ncAuPda ≈50 nm) was measured from FIB cross sections. The ncPda metal films were annealed in situ for ≈1 min at 300 °C and ≈18 min at 300 °C plus an additional ≈22 min at 350 °C for ncAuPda (plus the cooling down time of the holder) using a Gatan TEM heating holder during continuous STEM imaging for a fine adjustment of the grain size. After the annealing step, the grain size and twin density was measured using ACOM-STEM (Supporting Information File 1, Table S1).

A FEI Strata 400S dual beam FIB was used to transfer the metal C films to the push-to-pull (PTP) device (Hysitron) (Supporting Information File 1, Figure S1c,d) and to cut the films using an acceleration voltage of 30 kV and a beam current of 980 nA. The final shaping of the dog bone straining sample was performed at 30 kV and 1.5 nA beam current.

A Philips Tecnai F20 ST TEM operated at 200 kV in µp-STEM mode using spot size 7, gun lens 6, an extraction voltage of 4.5 kV and a 50 µm C2 aperture and equipped with the NanoMEGAS ASTAR system was used for ACOM-STEM data acquisition. A camera length of 100 mm was used to acquire the diffraction patterns.

A Hysitron Picoindenter PI95 with a push-to-pull (PTP) device (Supporting Information File 1, Figure S1a,b) operated under load control (0.1 µN/s) was used for the in situ straining experiments and was operated similar as described in [37]. The displacement was continuously monitored by the live µp-STEM imaging until a load holding segment was reached, which was necessary to acquire the ACOM-STEM orientation maps (Supporting Information File 1, Figures S5 and S12). Before and after the loading ramps, high quality µp-STEM overview images were acquired as reference for determining the strain (all strain values in this paper are giving relative to the initial dog bone length) and the spring constant of the PTP device was measured with the film ruptured to subtract the PTP device related forces from the measured stress–strain curve (Supporting Information File 1, Figures S3 and S13) [52]. Digital image correlation and tracking (DICT) was used to measure the strain during the loading ramps [53]. DICT was done twice per data set, once on the edges of the PTP to subtract the force coming from the PTP spring, and once on the edges of the dog bone to reveal the strain close to the area of interest.

The acquired ACOM-STEM data has been processed by the evaluation routine described in [37] to reduce the noise and to track crystallites through the straining series for the analysis of crystallite rotation, grain growth and twin activity. The evaluation routine used here differs from the one cited only in the noise filtering. Two new noise filters were developed: an “ambiguity filter” and a “minimum distance filter” [54]. The data processing parameters can be found in Supporting Information File 1.

## Supporting Information

File 1 Additional experimental results.
