# Supplementary material for: In situ observation of deformation processes in nanocrystalline face-centered cubic metals
Source: Beilstein J Nanotechnol. 2016 Apr 19;7:572–80. doi: 10.3762/bjnano.7.50 (PMC4901548; doi:10.3762/bjnano.7.50)
Supplement: File 1 — Additional experimental results. [file Beilstein_J_Nanotechnol-07-572-s001.pdf]

Supporting Information

for

In situ observation of deformation processes in nanocrystalline face-centered cubic metals

Aaron Kobler[1,2], Christian Brandl[3], Horst Hahn[1,2], and Christian Kübel[1,4*§]

Address: [1]Institute of Nanotechnology (INT), Karlsruhe Institute of Technology (KIT), Hermann-von-Helmholtz-Platz 1, 76344 Eggenstein-Leopoldshafen, Germany, [2]Joint Research Laboratory Nanomaterials (KIT and TUD), Technische Universität Darmstadt (TUD), Jovanka-Bontschits-Straße 2, 64287 Darmstadt, Germany, [3]Institute for Applied Materials (IAM-WBM), Karlsruhe Institute of Technology (KIT), Hermann-von-Helmholtz-Platz 1, 76344 Eggenstein-Leopoldshafen, Germany, and [4]Karlsruhe Nano Micro Facility (KNMF), Karlsruhe Institute of Technology (KIT), Hermann-von-Helmholtz-Platz 1, 76344 Eggenstein-Leopoldshafen, Germany

Email: Christian Kübel - christian.kuebel@kit.edu

* Corresponding author

[§]Tel.: +49 721 608 28970

Additional Experimental Results

Evaluation parameters for the processing of the ACOM-STEM orientation maps

The software versions used were: IMOD v4.6.0 [1], Mtex v3.5 [2,3], and ASTAR Index v1.3.4. The parameters for diffraction pattern simulation generation were as follows: maximum angle 3.5, excitation error 1, step count 50, intensity scale 1, minimum intensity 0.1; and for orientation map generation through template matching: smoothing radius 5, center shift 5, softening loop 3, enhancement loop 3, spot radius 5, noise threshold 20, gamma 0.33, normalization ≈0.6, fast matching using 20 test counts. Other parameters lead to slightly different orientations of few pixel. However, it does not affect the statement of this paper.

Quantitative evaluation of orientation maps

Ambiguity filter and minimum distance filter with first neighbors and weighting by the product of cross correlation index and reliability was applied [4]. A disorientation angle between the neighboring crystallites was set to 3° for crystallite segmentation. Grains where less than 50% of the pixels had a cross correlation index better than 20 or a reliability better than 0.15 were removed. Grains with an area smaller than 15 pixels and crystallites with an area smaller than 15 pixel were removed. Coincidence site lattice (CLS) Σ3 and Σ9 boundaries were detected based on the Ishida and McLean criteria [5] (±5°; ±1.7°, respectively; using the Brandon criteria [5] the tolerance angle is (blue ±8.7°; red ±5°, respectively). Crystallites that were detected as ambiguity crystallites were not plotted in rotation maps (Figures 4 and 5) or taken into consideration for the rotation histogram (Figure 6). For the ambiguity detection of crystallites between the orientation maps (Figure 6) the orientation map *i* was

compared to the map i+1. This reduces the error of ambiguity detection due to bending or tilting of the film. Rotations smaller than 5° are not plotted in Figure 6.

Straining of a carbon film

In the straining experiment a carbon film was used to support the nc sample. Therefore, the stress–strain curve of the nc sample is overlaid with the force coming from the C-film. To estimate the force contribution from the C-film to the total force of the metal-C-film a tensile experiment was conducted on the C-film alone (Figure S22).

The preparation of the dog bone sample and the conduction of the tensile experiment was done analog to ncPd[a] and ncAuPd[a]. The chemical composition after the preparation was checked by EELS to be C(87.6a-%), O(8.7a-%) and Cu(3.7a-%). The Cu comes from the Cu frame, that was used to transfer the thin film to the PTP device and was cut after the transfer (Figure S22a, Figure S1c,d). The thickness was determined out of EF-TEM thickness maps to be 34 nm in the inner part of the doge bone and 15 nm at the outer parts taking the chemical composition into consideration. To display the stress–strain curve the cross section in the smallest part of the dog bone was determined (Figure S22c). The experiment reached its methodical limits at ≈11% strain. The stress increases continuously till this strain as it is expected for a polymer film.

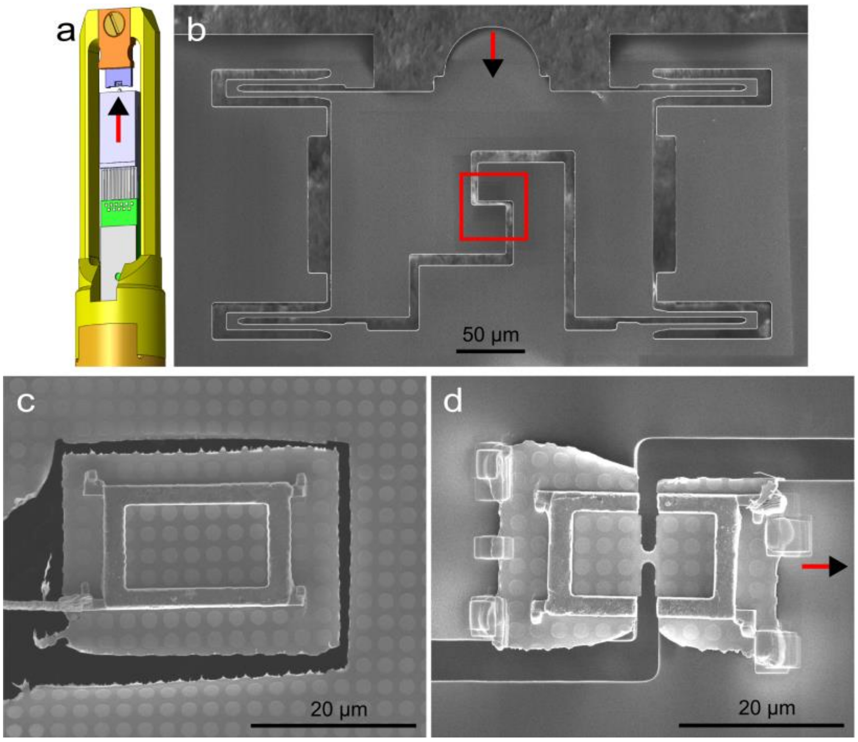

Figure S1: Straining setup using the Picoindenter (a) and the PTP devices (b) (Hysitron). Magnetron sputtered samples were transferred to the PTP with the FIB using a frame to stabilize the sample during the transfer (c). d) shows the sample after the transfer and after the cutting of a dog bone shape for tensile testing. Red square in (b) shows the area where the sample sits on the PTP.

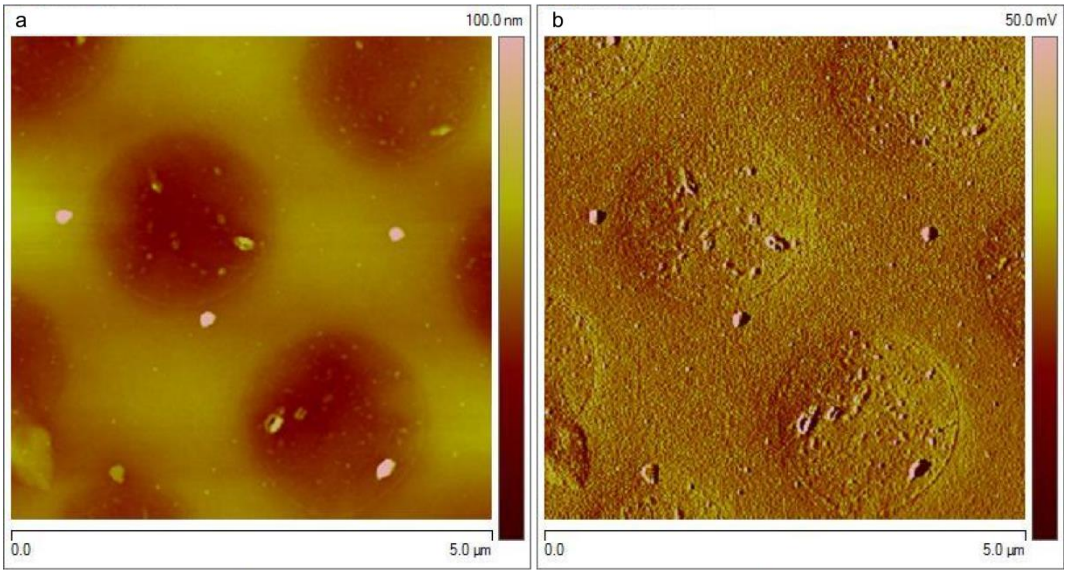

Figure S2: AFM measurement of magnetron sputtered Pd on holy carbon films before annealing: AFM height profile (a) and amplitude (b).

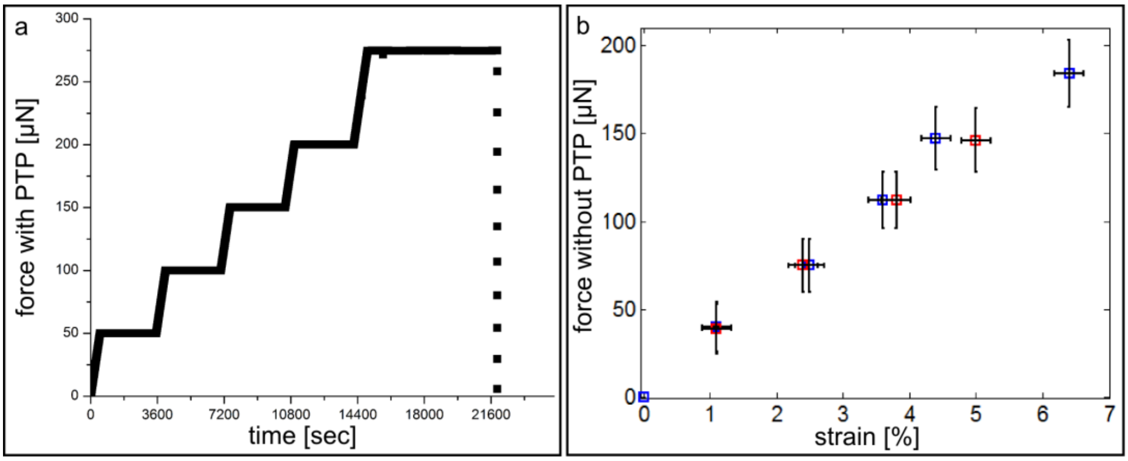

Figure S3: Straining curves of ncPd[a]: a) Force-time curve (Picoindenter output). The force includes the PTP force. b) Force-strain-curve after the subtraction of the PTP force contribution. The strain is derived from STEM images of the PTP gap and the length of the sample. The force of the PTP contribution is derived from a straining experiment of the PTP after rupture of the film. The blue points correspond to the measurements before ACOM-STEM acquisition, the red points the ones after ACOM-STEM acquisition. The error on the strain is taken from the uncertainty of the length measurement from the STEM images and the force error comes from the uncertainty of the force constant determination of the PTP.

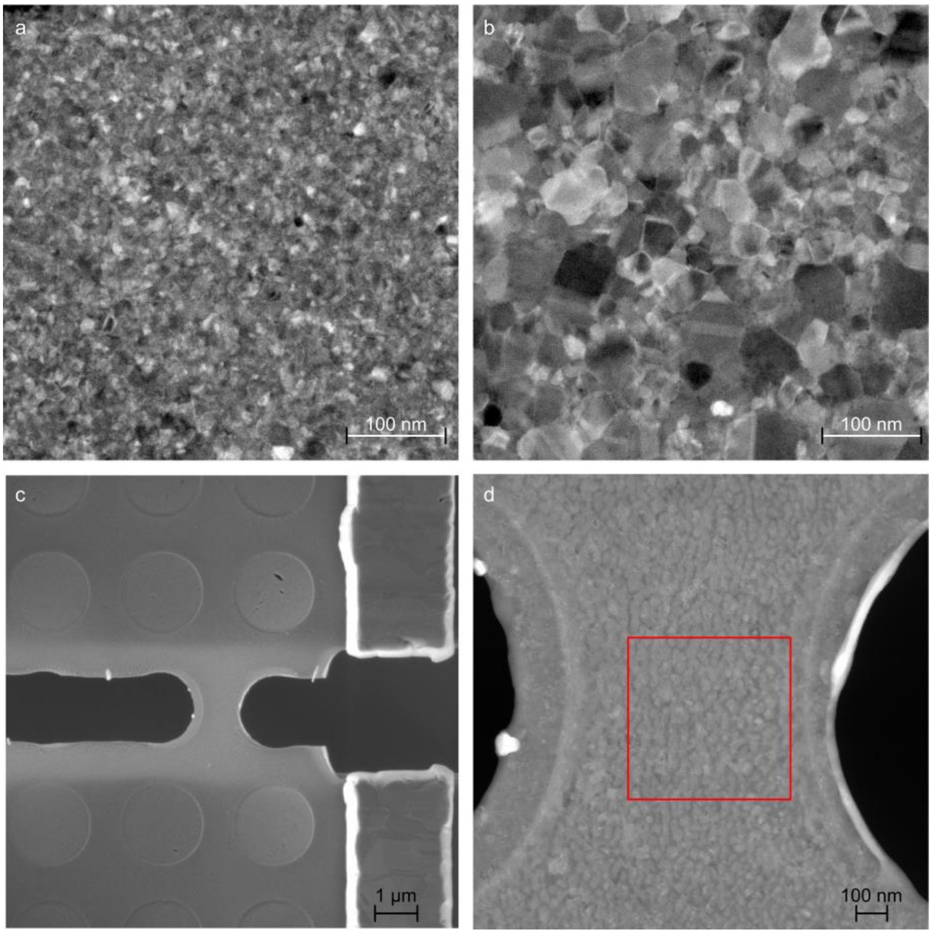

Figure S4: STEM overview of the straining sample: a) As sputtered ncPd[a] film. b) After in-situ heating inside the TEM. c) Dogbone sample prepared from the annealed ncPd[a] thin film using the FIB preparation shown in Figure S1. d) Zoom in onto the dogbone sample. The red square indicates the location where the orientation maps are acquired from.

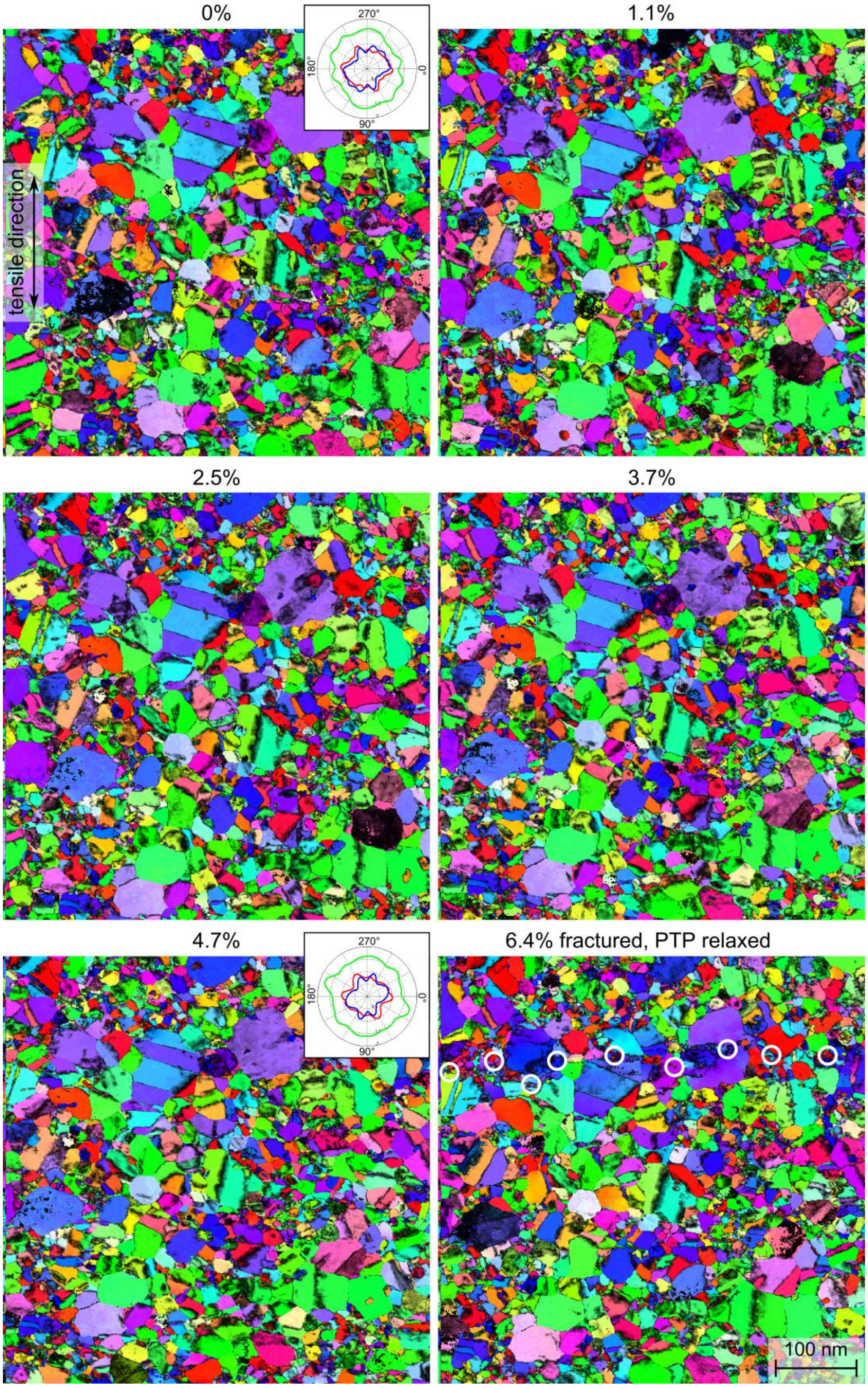

Figure S5: ACOM-STEM orientation maps of ncPd[a] overlaid with the reliability for all straining states till the fracturing of the sample. The projection direction is along the tensile direction. Insets at 0% and 5.4% strain show the in-plane texture plot. The color code is given in Figure 1c. White rings mark the crack site after fracturing of the film and the relaxation of the PTP that drives the fractured pieces back together.

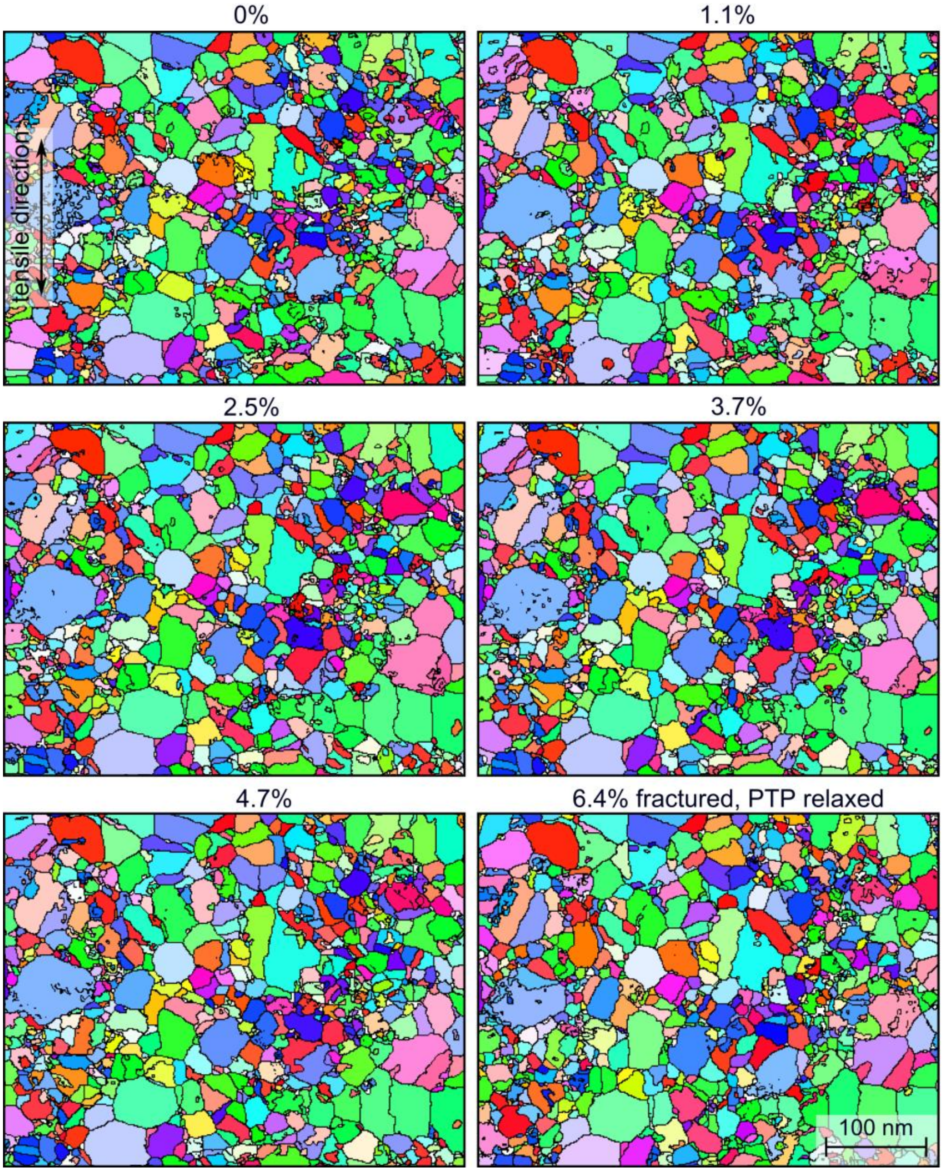

Figure S6: ACOM-STEM orientation maps of ncPd[a] after filtering and grain recognition for all straining states. The projection direction is along the tensile direction and the color code is given in Figure 1c.

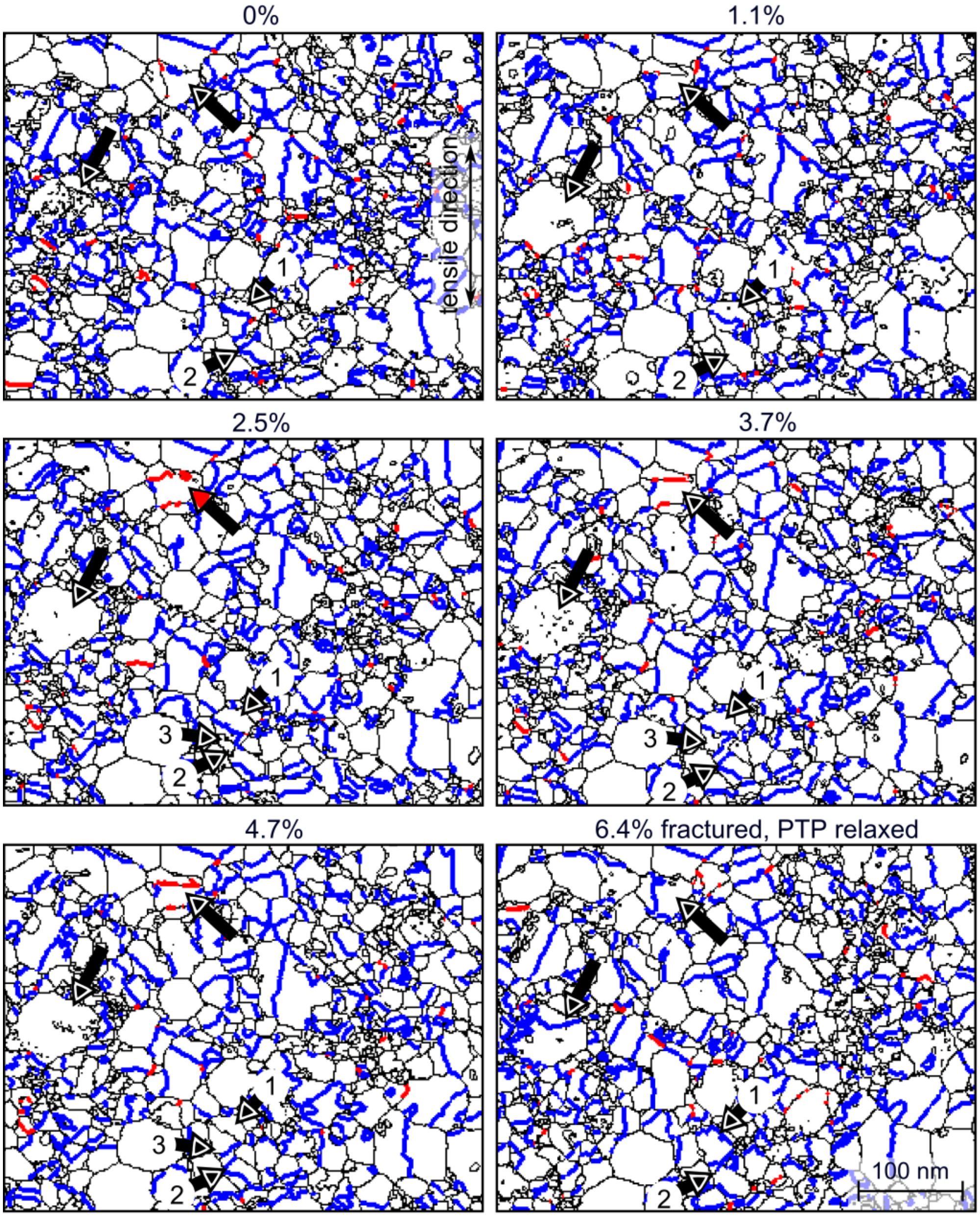

Figure S7: ACOM-STEM crystallite boundary maps of ncPd[a] after filtering and grain recognition for all straining states (black= crystallite boundaries, blue = Σ3 boundaries, red = Σ9 boundaries). Arrows indicate changes during the tensile experiment.

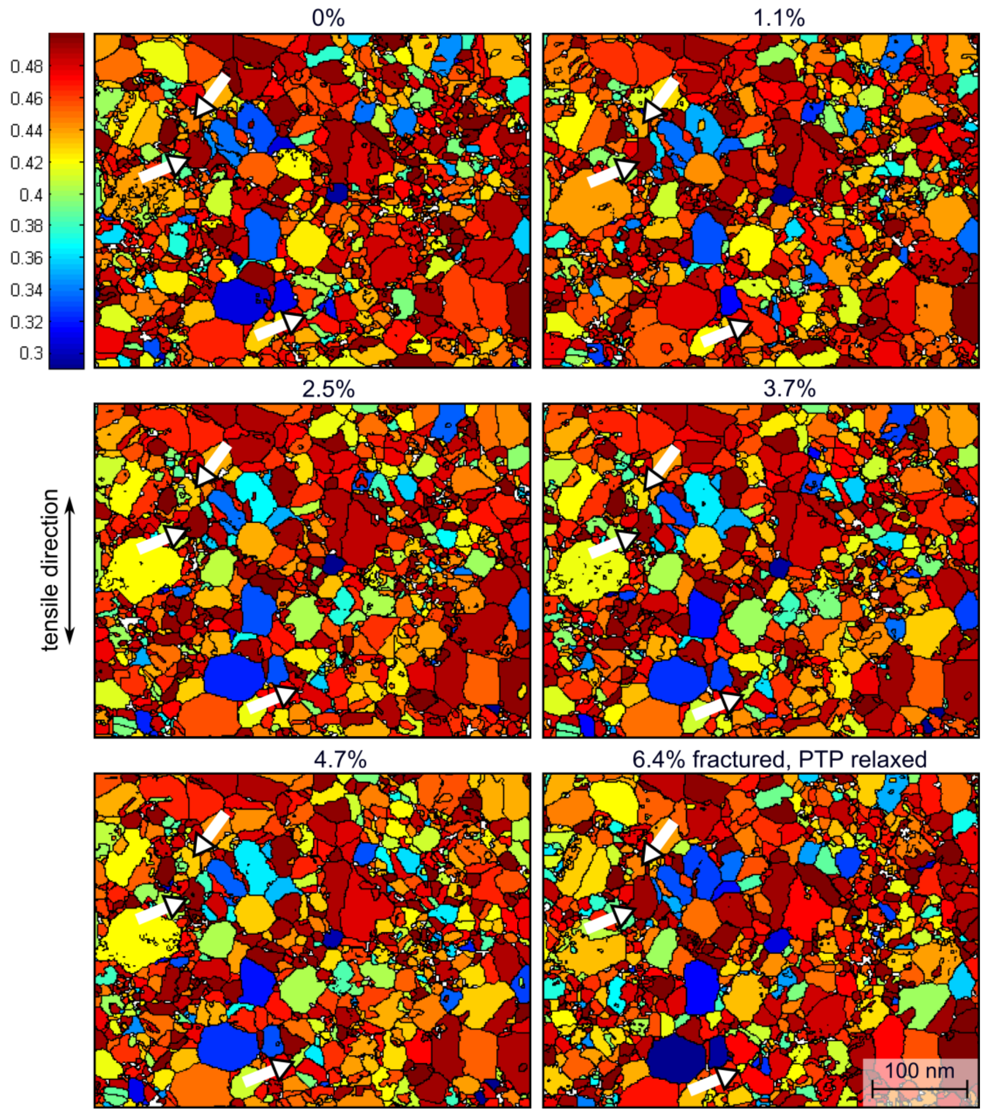

Figure S8: Filtered Crystallites of ncPd[a] colored with the Schmid Factors (An uniaxial load tensor was used). The arrows point at the crystallites highlighted in Figure 2,3.

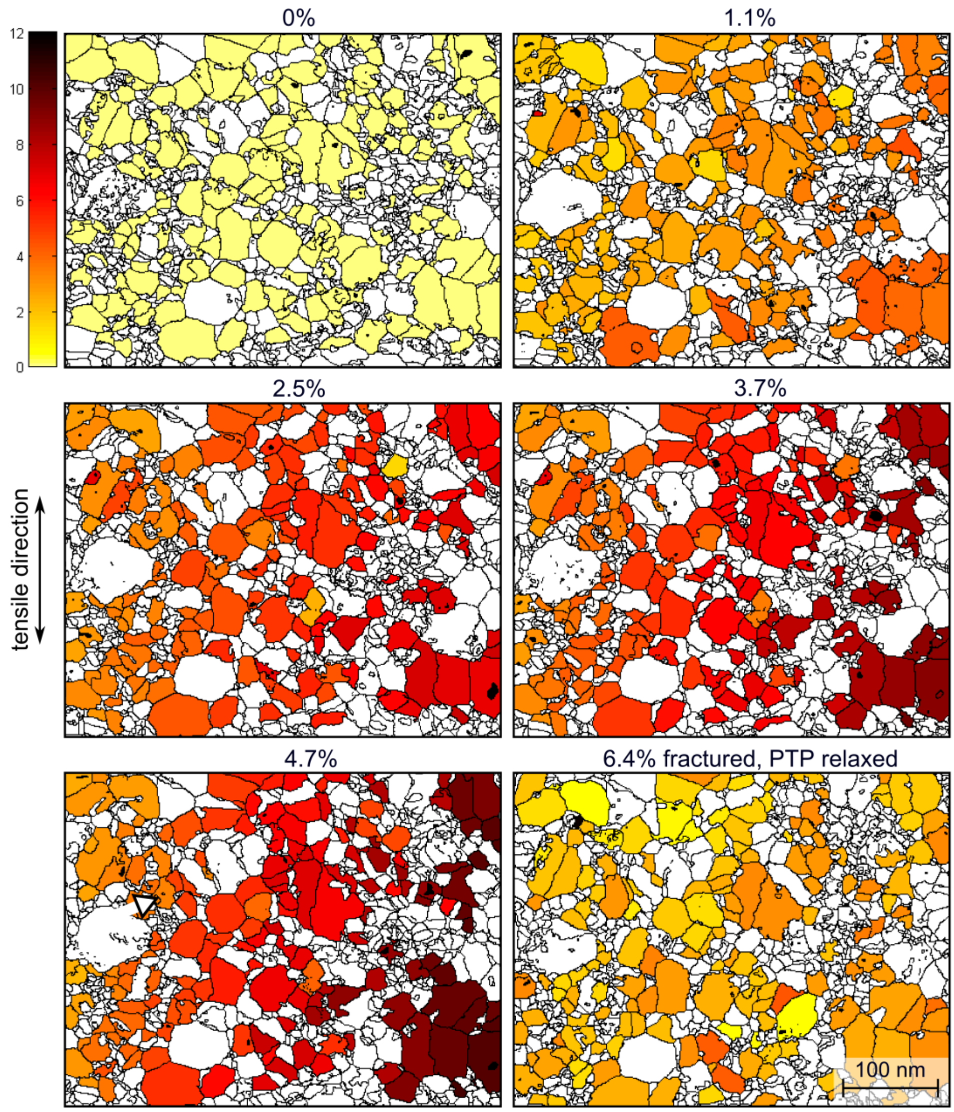

Figure S9: Filtered Crystallites of ncPd[a] colored with the absolute Rotation. Scale bar set to 12°.

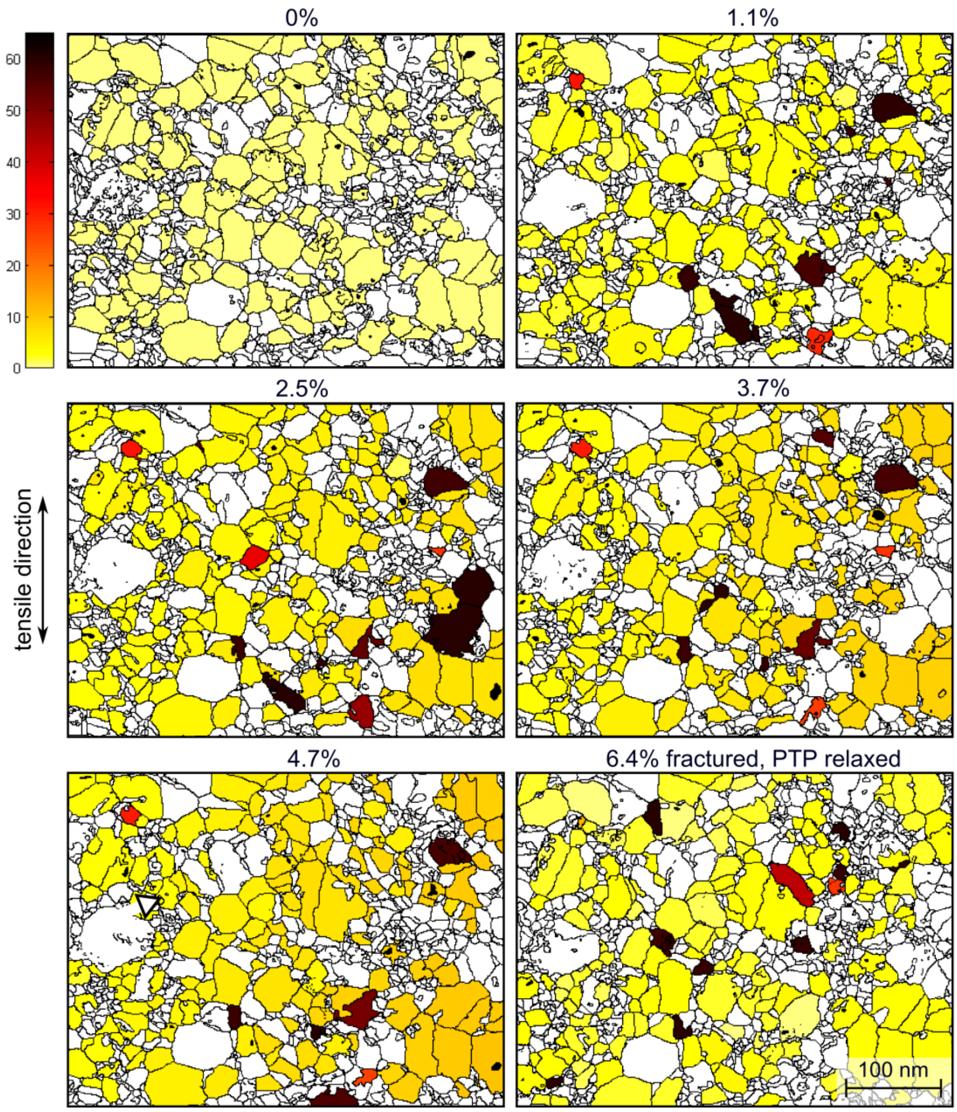

Figure S10: Filtered Crystallites of ncPd[a] colored with the absolute Rotation. Scale bar set to 65°.

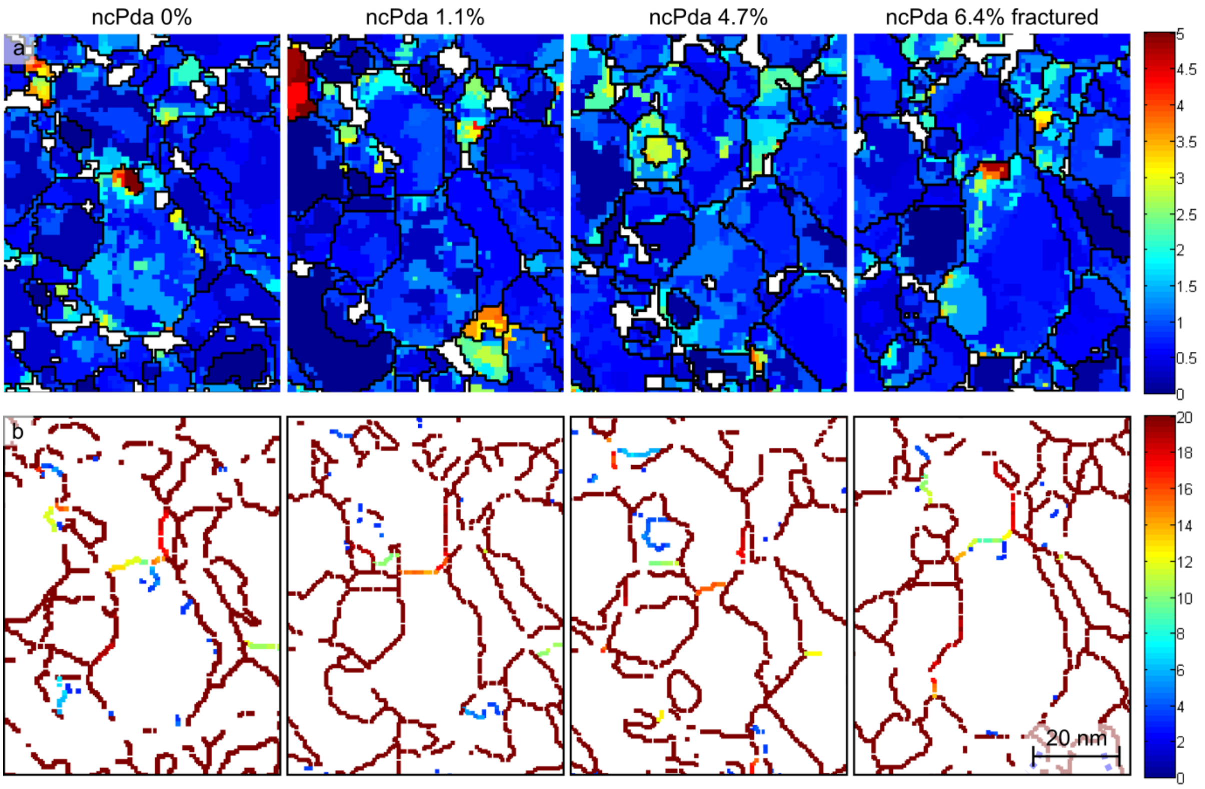

Figure S11: Detailed analysis for Figure 2: a) Misorientation to mean orientation of crystallites plot for selected straining states. b) Boundary disorientation plot. Color coding is given to the right correspondingly.

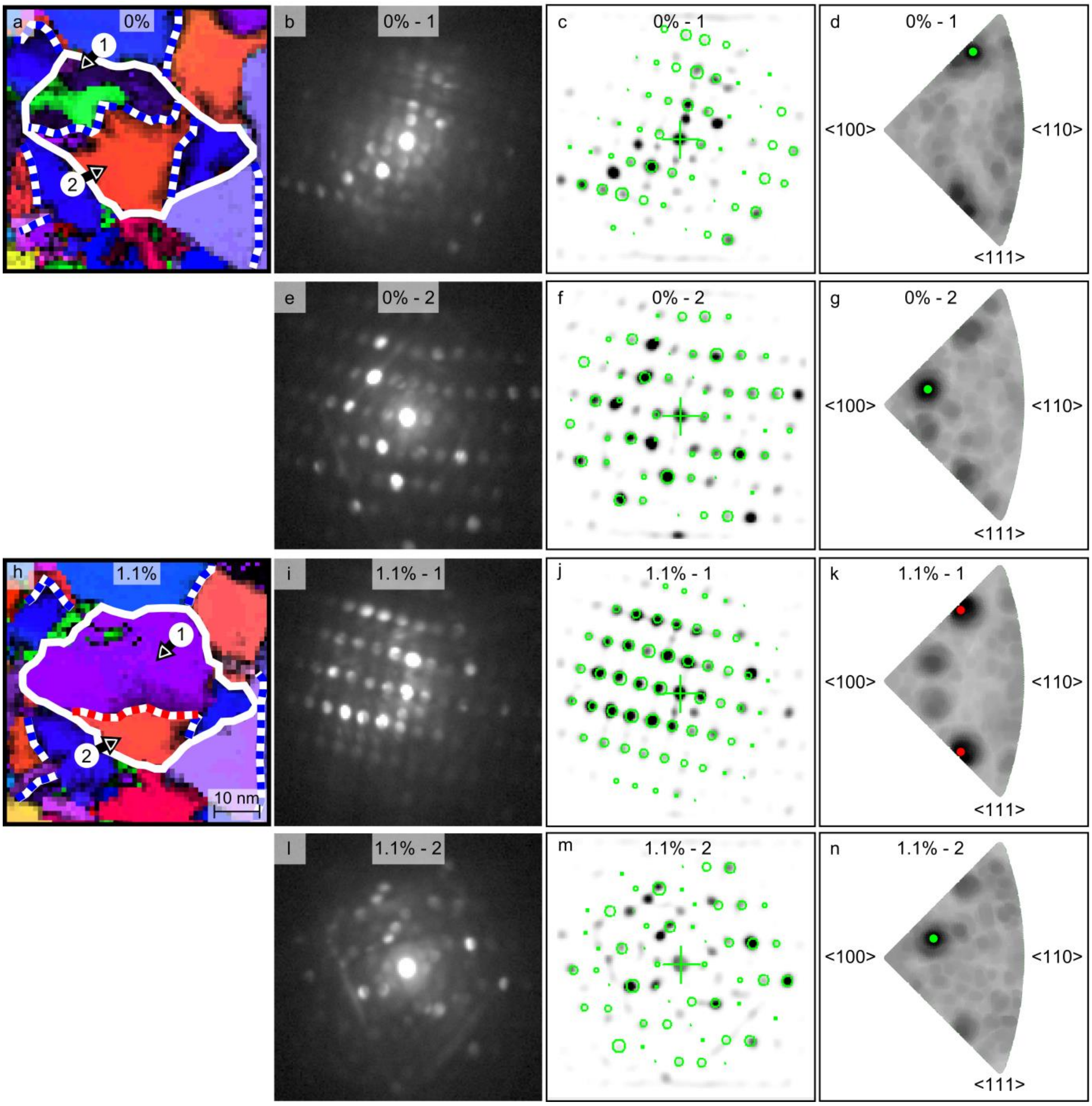

Figure S12: Addition to Figure 4b: Detailed comparison of a twinning process within a grain of ncPd[a]: a-g) 0% strain; h-n) 1.1% strain; a, h) ACOM-STEM orientation maps overlaid with the reliability (Σ3 disorientations marked with white/blue dashed lines and Σ9 disorientations marked with white/red dashed lines). Two areas of the marked grains (white line) of both straining states are analyzed; b,e,i,l) Experimental spot diffraction pattern of both areas correspondingly; c,f,j,m) Simulated diffraction pattern automatically matched the experimental diffraction patterns; d,g,k,n) Cross correlation index map for all crystal orientations in the representation of the inverse pole figure. The colored dots of the graphs

mark the crystal orientation with the highest cross correlation index of the given areas within the grains. The red dots of (k) mark the ambiguity orientation inside area 1. The disorientation between the two areas of 0% straining is ~60°, and that of 1.1% is ~40°. The disorientation between area 1 of both straining states is ~60° and that of area 2 is 2-3°. If the overlapping area 1 of 0% straining is compared with area 2 of straining state 1.1% it reveals a disorientation of ~40°. Only area 1 of 0% straining has a reliability value of 18%, the rest have values above 30%.

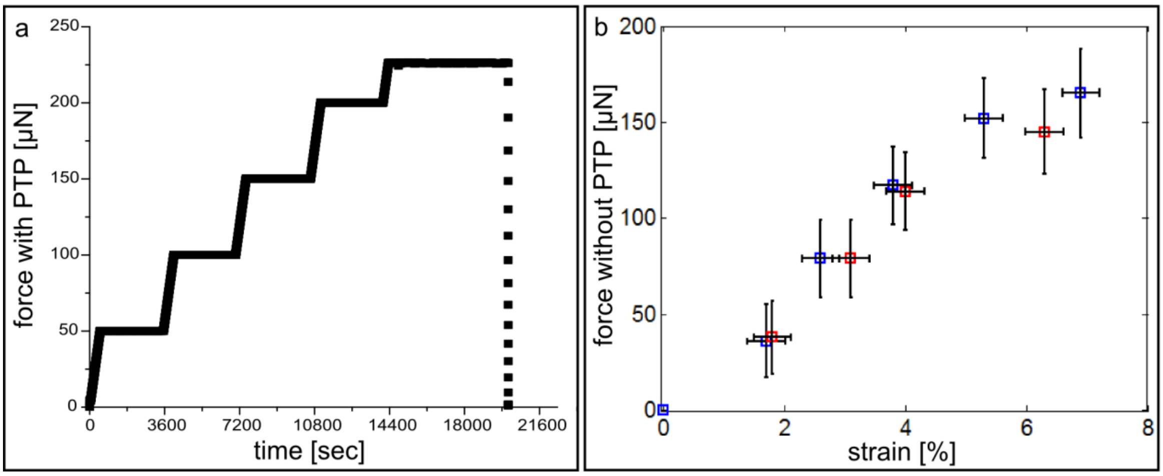

Figure S13: Straining curves of ncAuPd[a]: a) Force-time curve (Picoindenter output). The force includes the PTP force. b) Force-strain-curve after the subtraction of the PTP force contribution. The strain is derived from STEM images of the PTP gap and the length of the sample. The force of the PTP contribution is derived from a straining experiment of the PTP after rupture of the film. The blue points correspond to the measurements before ACOM-STEM acquisition, the red points the ones after ACOM-STEM acquisition. The error on the strain is taken from the uncertainty of the length measurement from the STEM images and the force error comes from the uncertainty of the force constant determination of the PTP.

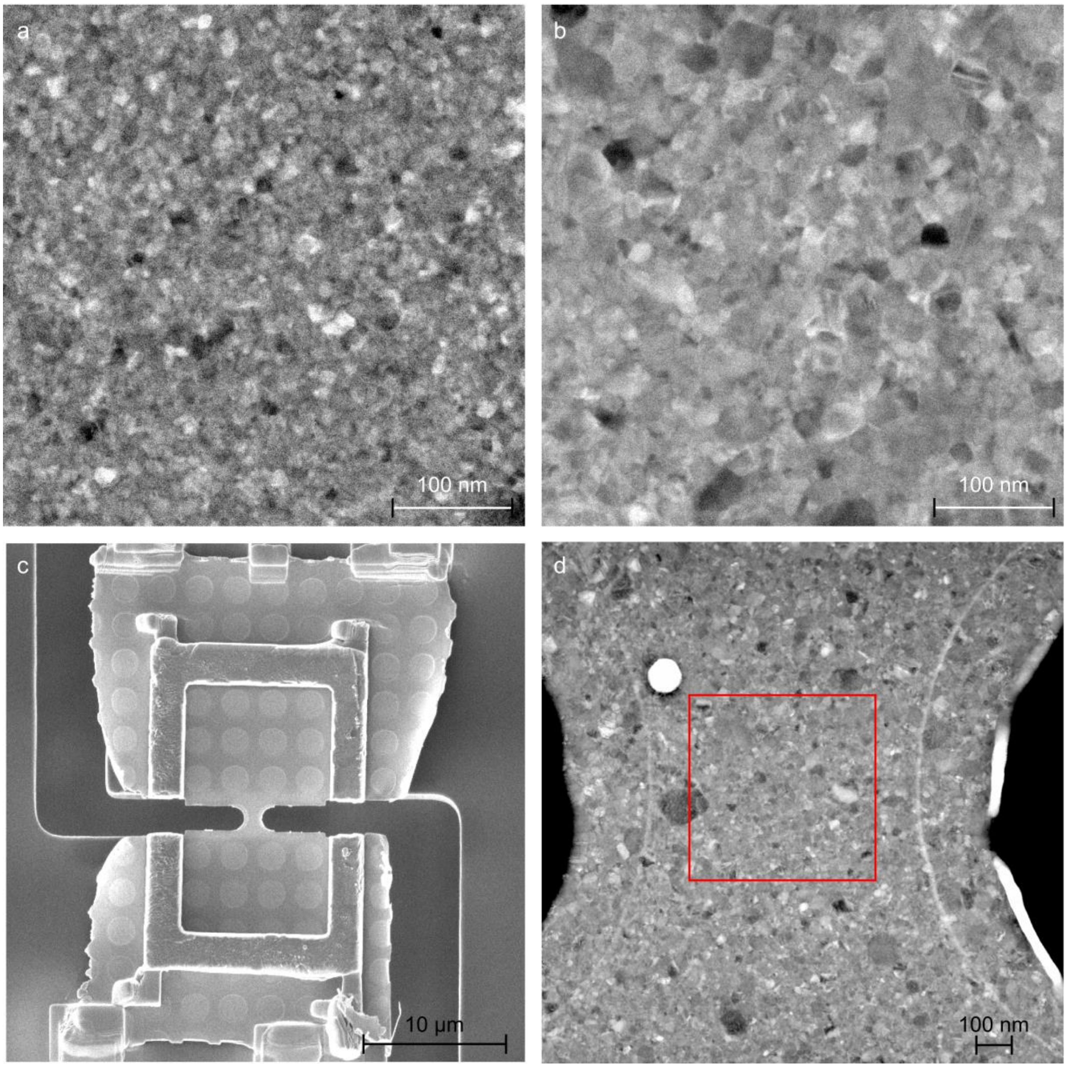

Figure S14: STEM overview of the straining sample: a) As sputtered ncAuPd[a] film. b) After in-situ heating inside the TEM. c) Dogbone sample prepared from the annealed ncAuPd[a] thin film using the FIB preparation shown in Figure S1. d) Zoom in onto the dogbone sample. The red square indicates the location where the orientation maps are acquired from.

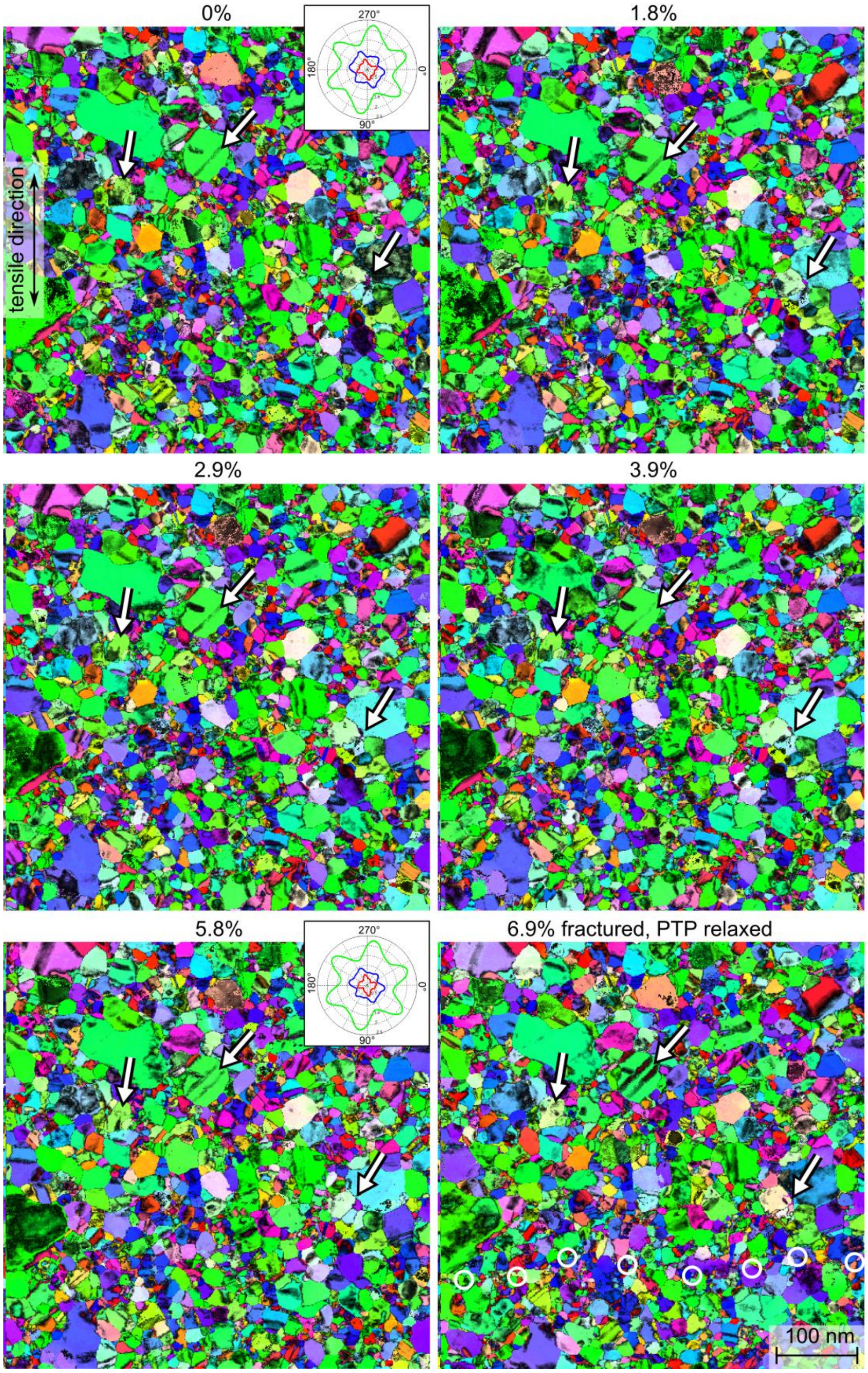

Figure S15: ACOM-STEM orientation maps of ncAuPd[a] overlaid with the reliability for all straining states till the fracturing of the sample. The projection direction is along the tensile direction. Insets at 0% and 5.8% strain show the in-plane texture plot. The color code is given in Figure 1c. White rings mark the crack site after fracturing of the film and the relaxation of the PTP that drives the fractured pieces back together.

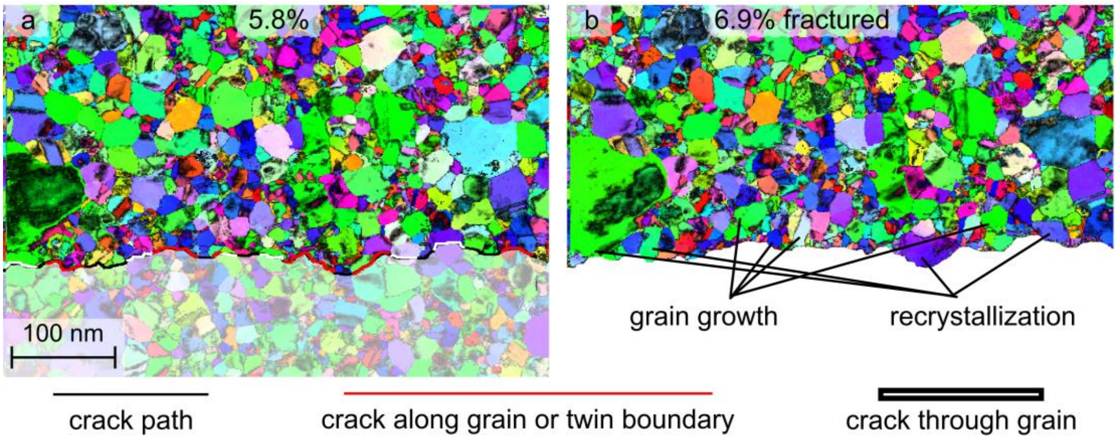

Figure S16: a,b) ACOM-STEM orientation maps overlaid with the reliability of the fracture morphology of ncAuPd[a] before (a) and after fracture (b). The crack line of (b) is overlaid in (a) to reveal the crack behavior. Red parts of the crack line indicate fracture along grains boundaries and white lines cracks passing through grains. The projection direction is along the tensile direction and the color code is given in Figure 1c.

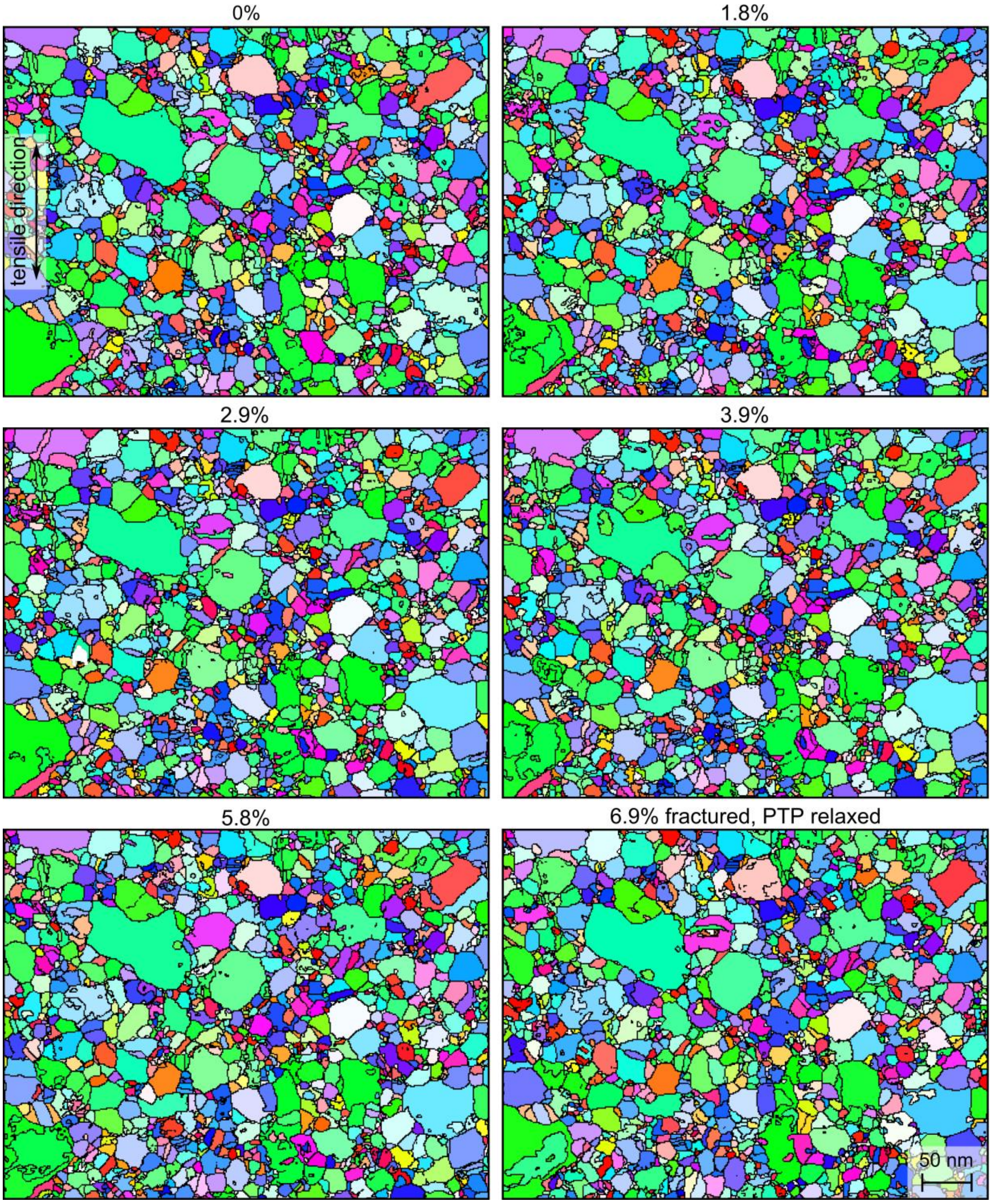

Figure S17: ACOM-STEM orientation maps of ncAuPd[a] after filtering and grain recognition for all straining states. The projection direction is along the tensile direction and the color code is given in Figure 1c.

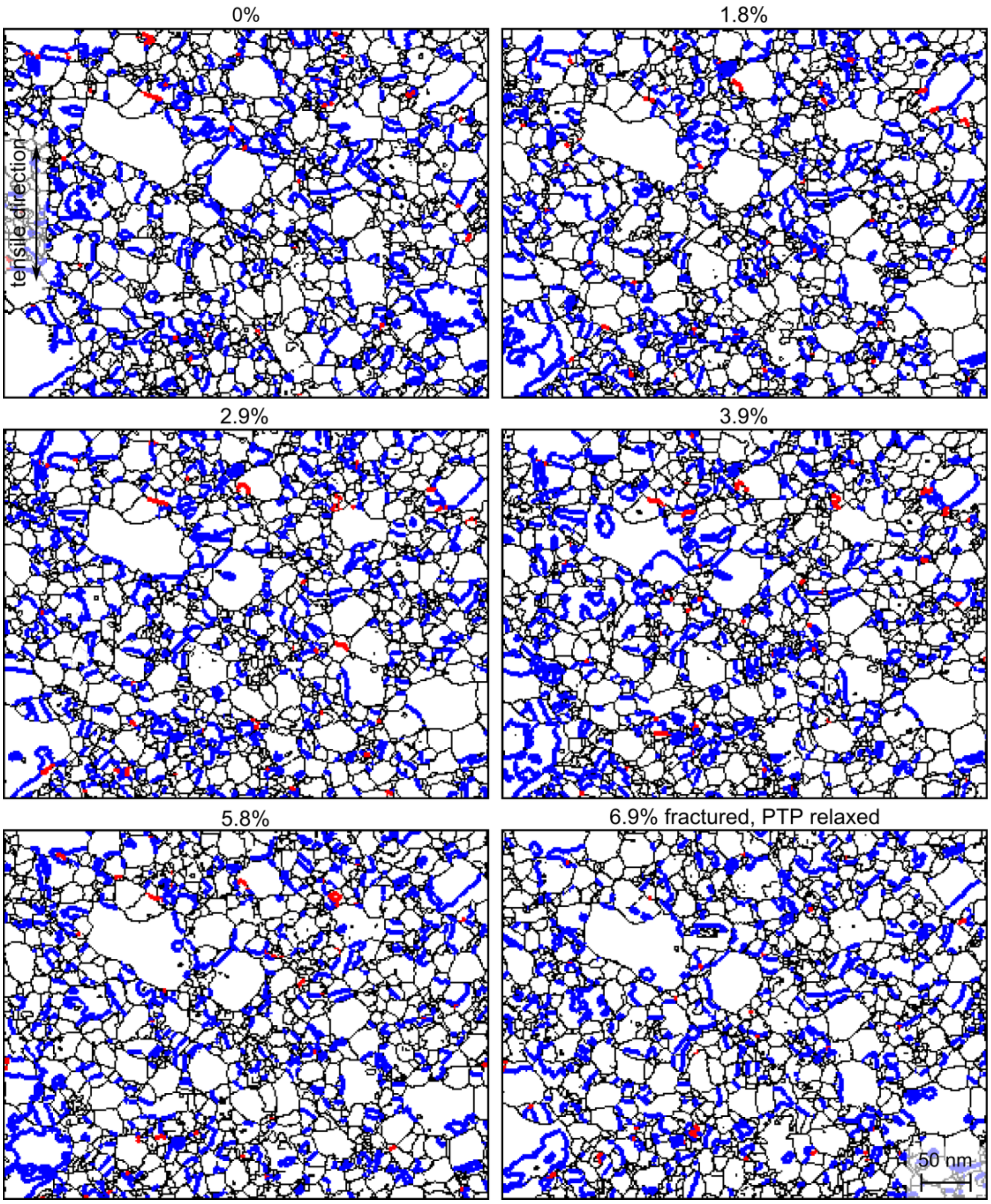

Figure S18: ACOM-STEM crystallite boundary maps of ncAuPd[a] after filtering and grain recognition for all straining states (black= crystallite boundaries, blue= Σ3 boundaries, red= Σ9 boundaries). Arrows indicate changes during the tensile experiment.

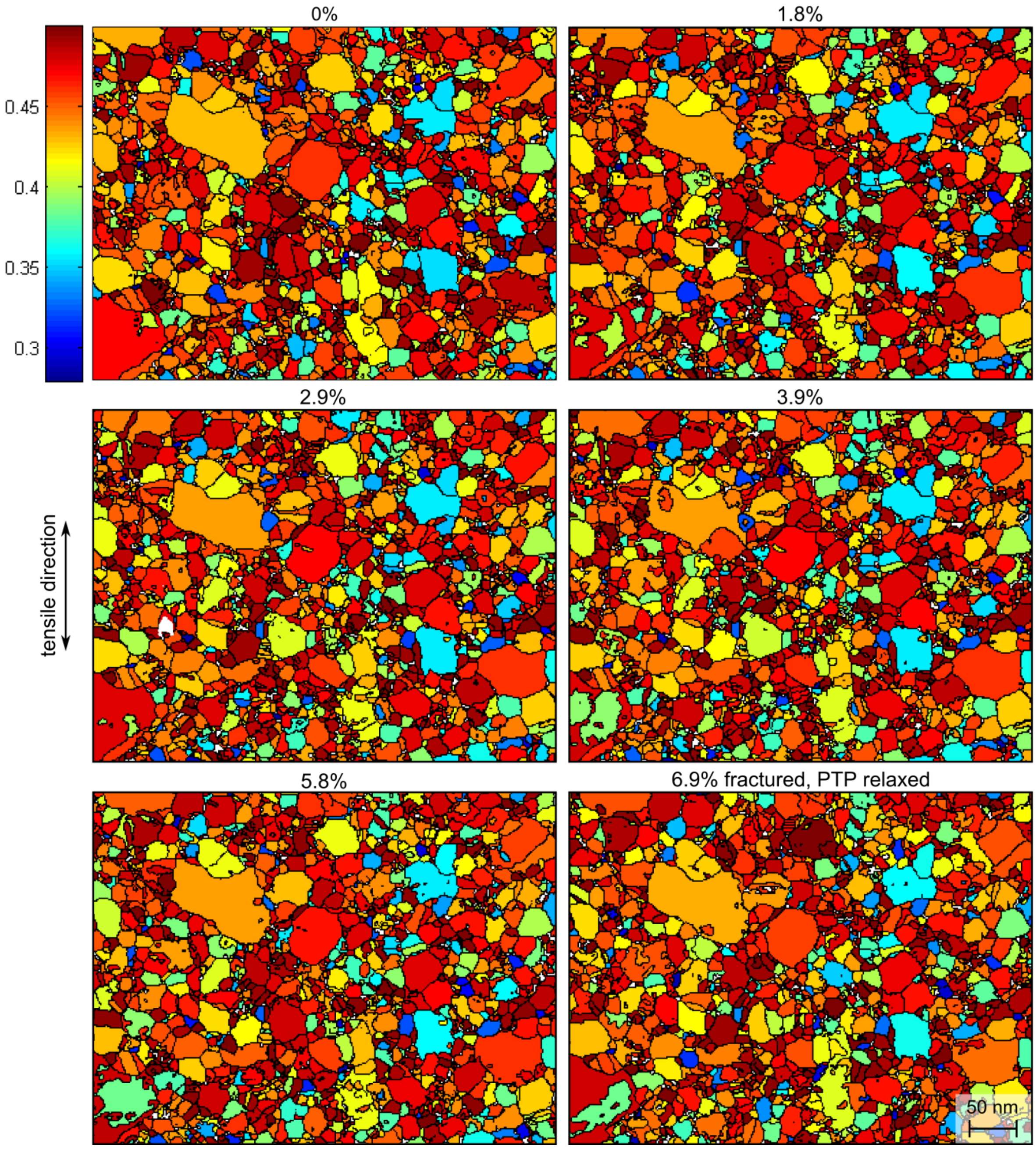

Figure S19: Filtered Crystallites of ncAuPd[a] colored with the Schmid Factors (An uniaxial load tensor was used).

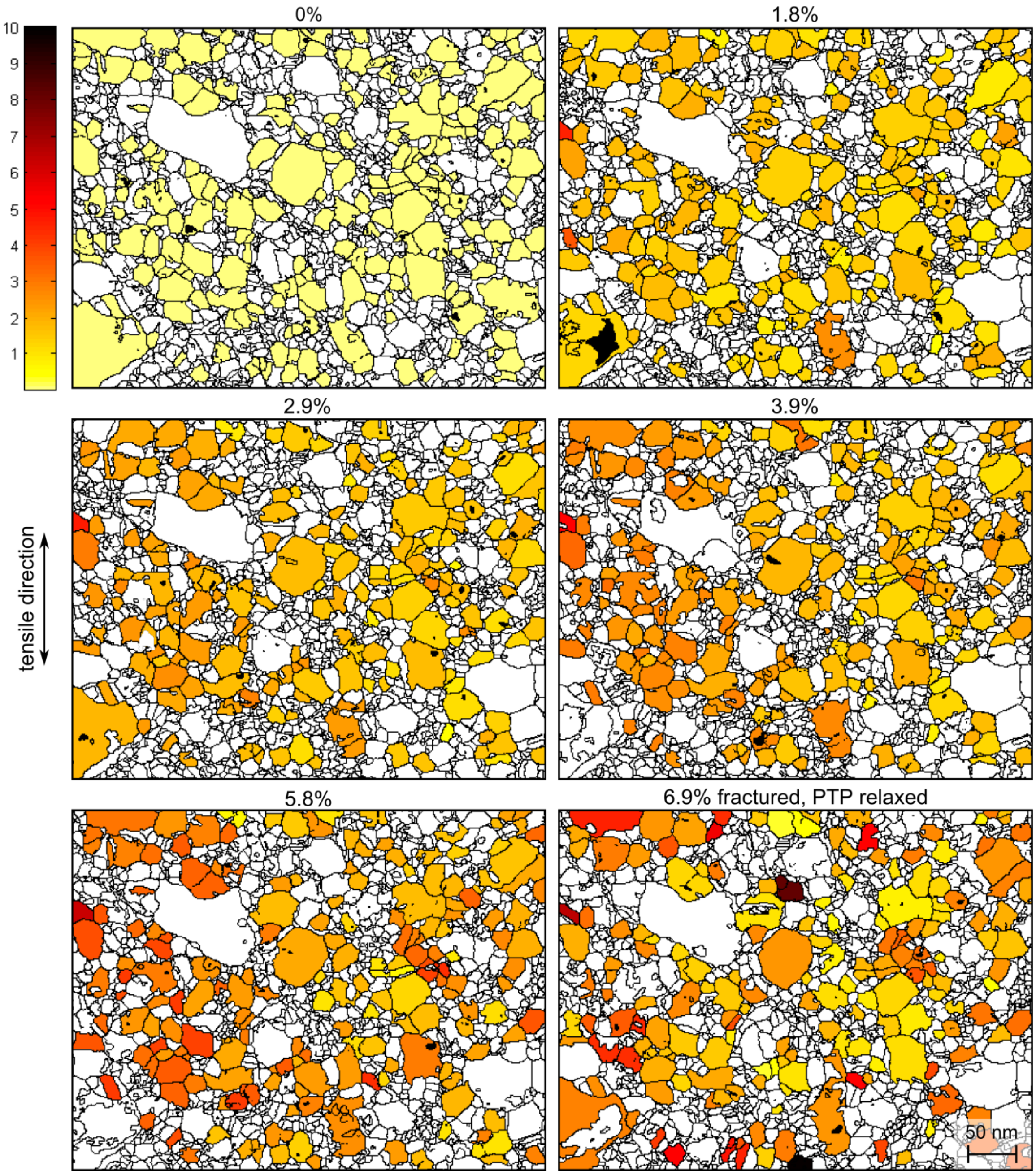

Figure S20: Filtered Crystallites of ncAuPd[a] colored with the absolute Rotation. Scale bar set to 10°.

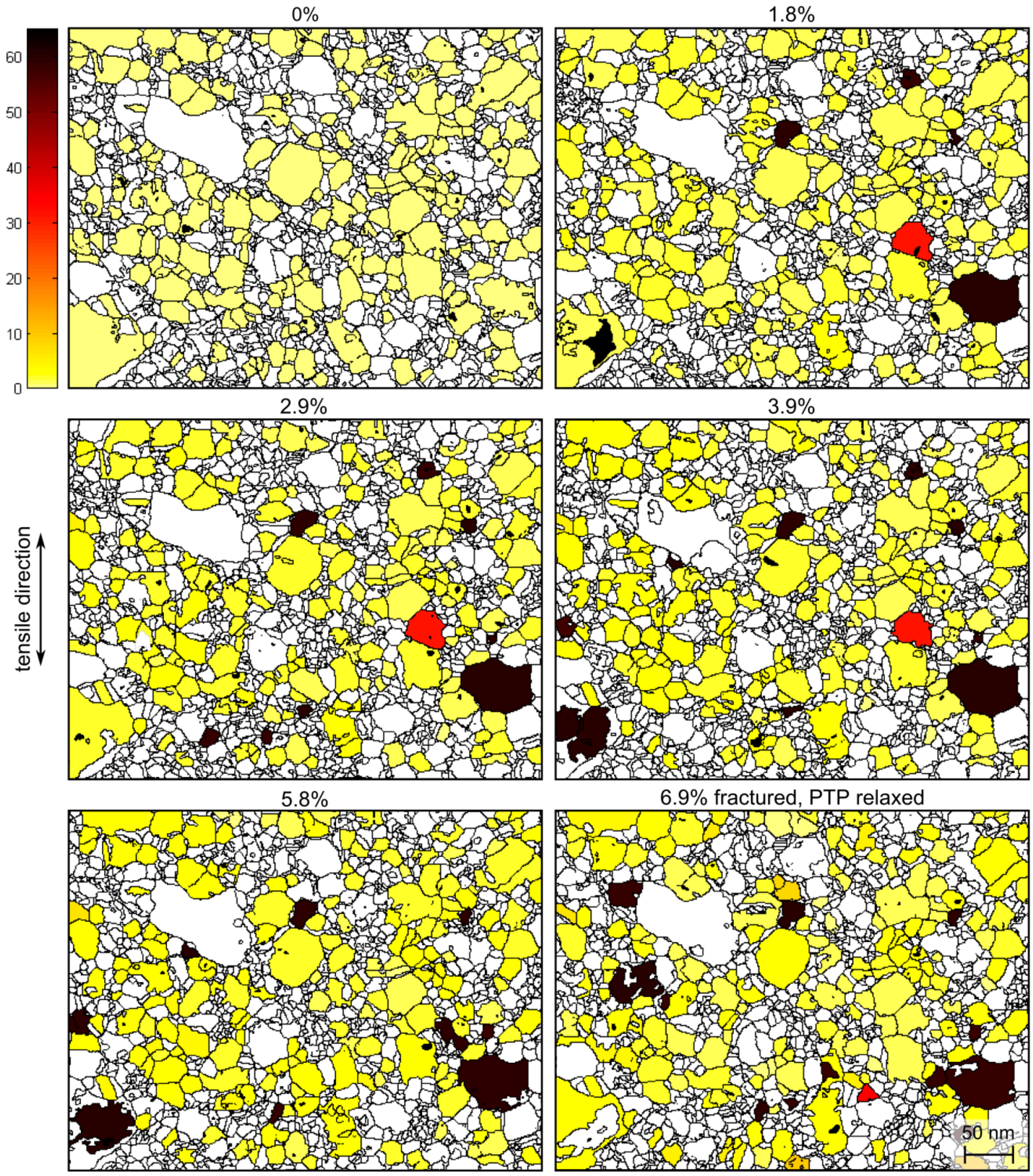

Figure S21: Filtered Crystallites of ncAuPd[a] colored with the absolute Rotation. Scale bar set to 65°.

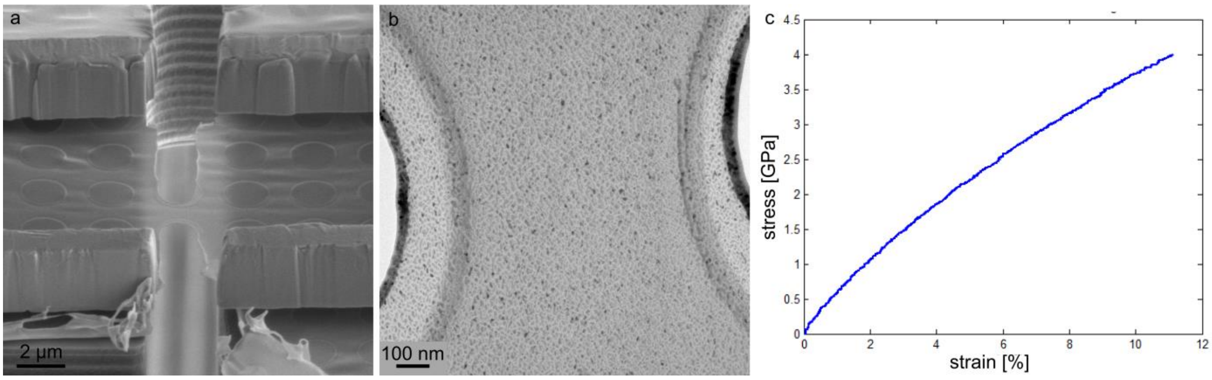

Figure S22: Straining experiment of a holy carbon film (Quantifoil) using the same technique as in Figure S1. a) Side view onto the dogbone sample of a holy carbon film. b) Top view of the dogbone sample. Black dots come from the preparation of the dogbone sample. c) stress-strain curve of the holy carbon film.

Table 1: Crystallite and grain size as well as twin density measured with ACOM-TEM in the orientation map at 0% strain for ncPd[a] and ncAuPd[a].

	average equivalent diameter				Twin density
	crystallites		grains		
	number average d_N [nm]	area average d_A [nm]	number average d_N [nm]	area average d_A [nm]	no. of twinned grains/ no. of total grains [%]
ncPd[a]	12	25	16	39	56
ncAuPd[a]	11	26	15	33	56

References

[1] Kremer, J. R.; Mastronarde, D. N.; McIntosh, J. R. Computer Visualization of Three-Dimensional Image Data Using IMOD. *J. Struct. Biol.* **1996**, *116*, 71–76.

[2] Bachmann, F.; Hielscher, R.; Schaeben, H. Texture Analysis with MTEX – Free and Open Source Software Toolbox. *Solid State Phenom.* **2010**, *160*, 63–68.

[3] Bachmann, F.; Hielscher, R.; Schaeben, H. Grain Detection from 2d and 3d EBSD Data--Specification of the MTEX Algorithm. *Ultramicroscopy* **2011**, *111*, 1720–1733.

[4] Kobler, A.; Kübel, C. Challenges in Quantitative Crystallographic Characterization of 3D Thin Films by ACOM-TEM. *Ultramicroscopy* (submitted).

[5] King, A. H.; Shekhar, S. What Does It Mean to Be Special? The Significance and Application of the Brandon Criterion. *J. Mater. Sci.* **2006**, *41*, 7675–7682.
